# Sevoflurane induces microRNA-18a to delay rat neurodevelopment *via* suppression of the RUNX1/Wnt/β-catenin axis

**DOI:** 10.1038/s41420-022-01179-y

**Published:** 2022-10-01

**Authors:** Yuge Jiang, Yaobo Liu, Yuhui Sun, Yongzhe Liu, Long Feng, Mingda Duan, Yi Liu, Longhe Xu

**Affiliations:** 1 Department of Emergency, The Second Medical Center of Chinese PLA General Hospital, Beijing, 100853 P. R. China; 2 Jiangsu Key Laboratory of Neuropsychiatric Diseases and Institute of Neuroscience, Soochow University, Suzhou, 215123 P. R. China; 3 Department of Anesthesiology, The Third Medical Center of Chinese PLA General Hospital, Beijing, 100853 P. R. China; 4 Department of Anesthesiology, Hainan Hospital of Chinese PLA General Hospital, Sanya, 5721000 P. R. China

**Keywords:** Adherens junctions, Diagnostic markers

## Abstract

Sevoflurane anesthesia is reported to repress neurogenesis of neural stem cells (NSCs), thereby affecting the brain development, but the underlying mechanism of sevoflurane on the proliferation of NSCs remains unclear. Thus, this study aims to discern the relationship between sevoflurane and NSC proliferation. Bioinformatics tools were employed to predict the expression of microRNA-18a (miR-18a) in 9-day-old neonatal rat hippocampal tissues after sevoflurane treatment and the downstream genes of miR-18a, followed by a series of assays to explore the relationship among miR-18a, runt related transcription factor 1 (RUNX1), and β-catenin in the hippocampal tissues. NSCs were isolated from the hippocampal tissues and subjected to gain-/loss-of-function assays to investigate the interactions among miR-18a, RUNX1, and β-catenin in NSCs and their roles in NSC development. Bioinformatics analysis and experimental results confirmed high expression of miR-18a in rat hippocampal tissues and NSCs after sevoflurane treatment. Next, we found that miR-18a downregulated RUNX1 expression, while RUNX1 promoted NSC proliferation by activating the Wnt/β-catenin signaling pathway. The behavioral experiments also showed that sevoflurane caused nerve injury in rats, whilst RUNX1 overexpression protected rat neurodevelopment. Our findings uncovered that sevoflurane attenuated NSC proliferation via the miR-18a-meidated RUNX1/Wnt/β-catenin pathway, thereby impairing rat neurodevelopment.

## Introduction

Anaesthetic agents are indispensable to relieve pain during childbirth or surgical operations since their first application, and sevoflurane is one of the most widely adopted volatile anaesthetics [1]. Sevoflurane plays the anaesthetic role by weakening the signal transduction between various brain regions which share specialization and functionality during consciousness [2]. However, a lot of research has underlined the serious neurotoxicity induced by sevoflurane and the harmful interaction of sevoflurane with neurodegenerative activities, such as the occurrence and progression of Alzheimer’s disease [3]. Another literature has indicated that exposure to high-concentration sevoflurane in the period of mid-gestation facilitates the apoptosis of neural stem cells (NSCs), the viability of which directly influences the brain development, in offspring rats [4]. However, the molecular mechanism of sevoflurane affecting the proliferation of NSCs is still elusive, which urgently requires further research.

Sevoflurane affects the expression of microRNAs (miRNAs or miRs), which are involved in the mediation of numerous cellular processes including proliferation and differentiation of NSCs [5, 6]. For instance, miR-137 enriched in the brain can control the dynamics between NSC proliferation and differentiation in the process of neurodevelopment [7]. Similarly, miR-18a is reported to suppress activation of several proliferation-related signaling pathways [8], and overexpressed miR-18a in human neuroblastoma can regulate neuronal differentiation [9], implying its implication in neurodevelopment. Further, our bioinformatics analysis has revealed that runt-related transcription factor 1 (RUNX1) is a putative downstream target gene of miR-18a. Mounting evidence shows that RUNX1 acts importantly in the modulation of the initial differentiation of adult neurogenesis, and after nerve injury, RUNX1 is regulated to facilitate NSC differentiation to promote repair of the complex nervous system [10, 11]. Intriguingly, a recent study has reported the ability of RUNX1 to directly affect β-catenin and drive activation of the Wnt/β-catenin signaling pathway [12], while the Wnt/β-catenin signaling pathway can regulate the differentiation of NSCs [13, 14]. It is worthy of noting that sevoflurane inhibits the proliferation and differentiation of NSCs by inhibiting β-catenin [15]. Given the above, we assumed that sevoflurane may exert a suppressive effect on NSC development through a miR-18a/RUNX1/Wnt/β-catenin pathway, which may give a reasonable explanation on the sevoflurane-induced neurotoxicity.

## Results

### miR-18a is highly expressed in rat hippocampal tissues and NSCs after sevoflurane treatment

**Fig. 1:**
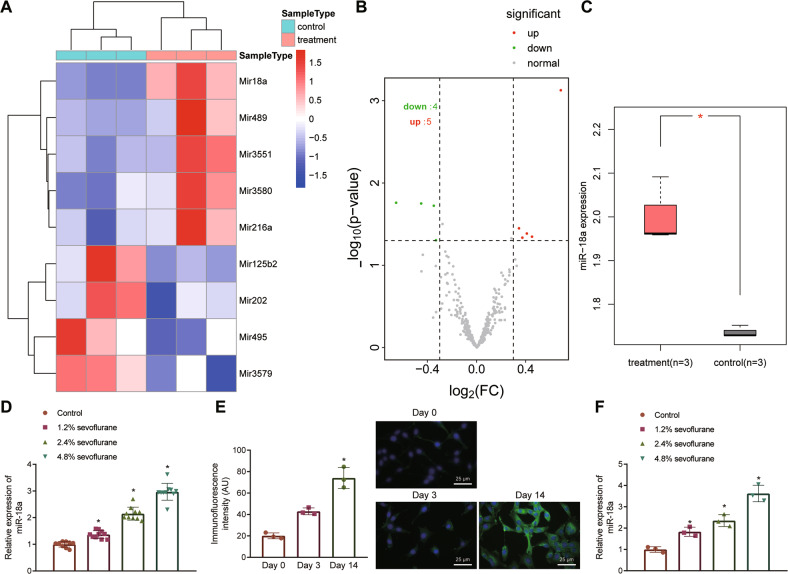
Sevoflurane treatment increases miR-18a expression in rat hippocampal tissues and NSCs. **A** Heat map of differentially expressed miRNAs in the microarray GSE141242 (control: *n* = 3; treatment: *n* = 3). **B** Volcano map of differentially expressed miRNAs in the microarray GSE141242. **C** Box plot showing the expression of miR-18a, wherein red indicates the sevoflurane treatment group (*n* = 3), gray indicates control group (*n* = 3) (*p < 0.05 vs. the control group). D, RT-qPCR detection of miR-18a expression in rat hippocampal tissues under different concentrations of sevoflurane treatment, *n* = 6, *p < 0.05 vs. the control group. E, Morphology of NSCs isolated 0, 3 and 14 d after primary culture and nestin protein expression under fluorescence microscope (*p < 0.05 vs. data at day 3) (bar = 25 μm); DAPI: the nucleus in blue fluorescence; nestin protein: green fluorescence. F, RT-qPCR detection of miR-18a expression in NSCs under different concentrations of sevoflurane treatment, *p < 0.05 vs. the control group. The results were measurement data, expressed as mean ± standard deviation, and analyzed by independent sample *t*-test. The cell experiment was repeated 3 times independently.

Sevoflurane is one of the most commonly used volatile anesthetics, and NSC differentiation ability directly affects brain development [4]. Microarray dataset GSE141242 (3 normal rats and 3 sevoflurane-treated rats) was retrieved from GEO for miRNA expression profile in rat hippocampal tissues after sevoflurane anesthesia, followed by differential analysis using limma package of R language. In our study, bioinformatics analysis showed that there were 9 differentially expressed miRNAs in rat hippocampal tissues after sevoflurane anesthesia (Fig. 1A), of which 5 miRNAs were upregulated and 4 miRNAs were downregulated (Fig. 1B), and the expression of miR-18a was most significantly different in rat hippocampal tissues after sevoflurane anesthesia (Fig. 1C).

Next, we divided 9-day-old neonatal rats into four groups (10 rats in each group): one group of rats was cultured normally in air (control group) for 6 h, and the other three groups of rats were exposed to sevoflurane at concentrations of 1%, 2%, and 3% (experimental groups) for 6 h. Hippocampal tissues were obtained from the four groups of rats to extract RNA. The results of reverse transcription-quantitative polymerase chain reaction (RT-qPCR) showed elevated miR-18a expression in rat hippocampal tissues after sevoflurane treatment with a dose-dependent trend (Fig. 1D). In addition, we isolated NSCs, and confirmed the positive expression of nestin protein (green fluorescent protein) in NSCs by immunofluorescence, indicating successful isolation of NSCs (Fig. 1E). At the same time, NSCs were treated with sevoflurane at concentrations of 1%, 2%, and 3%, and untreated NSCs were used as control. RT-qPCR depicted the same elevation of miR-18a expression in NSCs after sevoflurane treatment (Fig. 1F).

In addition, we isolated and obtained astrocytes and oligodendrocytes at the same time, and confirmed the positive expression of GFAP and O4 proteins (green fluorescent protein) in astrocytes and oligodendrocytes by immunofluorescence, respectively, indicating successful isolation of astrocytes and oligodendrocytes (Supplementary Fig. 1A). At the same time, astrocytes and oligodendrocytes were treated with 1%, 2%, and 3% sevoflurane, respectively, and untreated astrocytes and oligodendrocytes were used as controls. The results of RT-qPCR depicted that there was no significant change in the expression of miR-18a in astrocytes and oligodendrocytes treated with sevoflurane (Supplementary Fig. 1B). These results suggested that miR-18a was specifically highly expressed in NSCs after sevoflurane treatment.

Thus, miR-18a is elevated in rat hippocampal tissues and NSCs after sevoflurane treatment.

### Sevoflurane inhibits proliferation of NSCs and rat neurodevelopment by inducing miR-18a expression

**Fig. 2:**
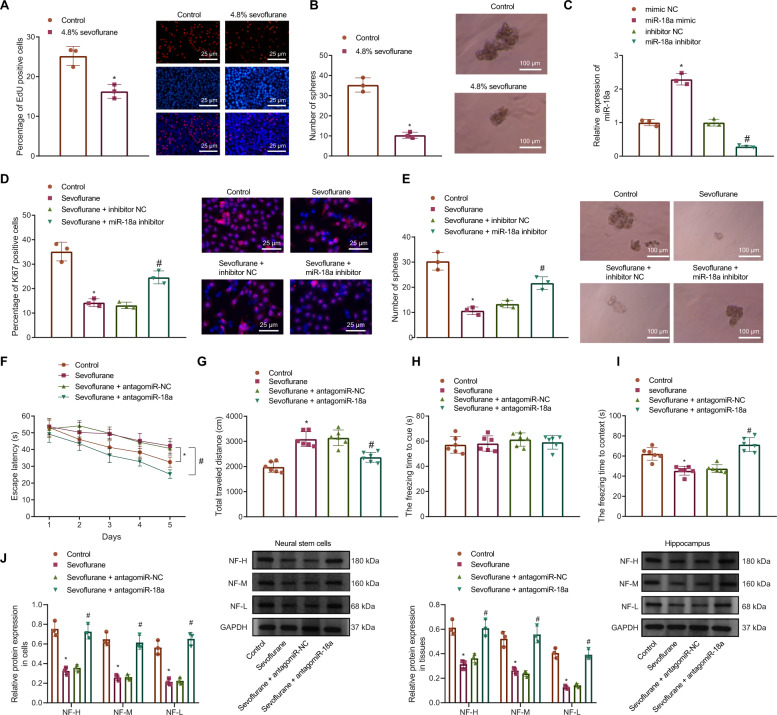
Sevoflurane induces miR-18a expression to affect NSC proliferation and neurodevelopment in rats. **A** The effect of 4.8% sevoflurane on the NSC proliferation detected by EdU staining (bar = 25 μm) (DAPI: the nucleus in blue fluorescence, EdU staining: red fluorescence). **B** The effect of 4.8% sevoflurane on the NSC sphere-forming ability (bar = 100 μm). **C** The efficiency of miR-18a knockdown or overexpression detected by RT-qPCR. **D** The effect of miR-18a expression on the proportion of Ki67 positive cells under sevoflurane treatment detected by immunofluorescence assay (bar = 25 μm); PI: the nucleus in red fluorescence, Ki67: green fluorescence. **E** The sphere-forming ability of NSCs after treatment of sevoflurane or miR-18a inhibitor (bar = 100 μm). **F** The effect of miR-18a expression under sevoflurane treatment on the movement time of rats in the water maze assessed by Morris water maze test; *n* = 6. **G** The effect of miR-18a expression under sevoflurane treatment on the movement distance of rats in the open field assessed by open field test; *n* = 6. **H** The effect of miR-18a expression on the freezing time to cue of rats under sevoflurane treatment assessed by conditioned fear test; *n* = 6. **I** The effect of miR-18a expression on the freezing time to context of rats under sevoflurane treatment assessed by conditioned fear test; *n* = 6. **J** The expression of neural cell marker proteins (NF-H/NF-M/NF-L) in cells and tissues after sevoflurane or antagomiR-18a treatment detected by Western blot analysis. *p < 0.05 vs. the control/mimic NC group, ^#^*p* < 0.05 vs. the inhibitor NC/sevoflurane + inhibitor NC/sevoflurane + antagomiR-NC group. Data were measurement data, expressed as mean ± standard deviation, and analyzed using independent sample *t*-test. The cell experiment was repeated 3 times independently.

To further explore the effect of sevoflurane on NSC growth, we used 3% sevoflurane for the treatment of NSCs in subsequent experiments. The 5-Ethynyl-2’-deoxyuridine (EdU) staining results showed that the proliferation rate of NSCs was significantly reduced after sevoflurane treatment (Fig. 2A). At the same time, it could be seen from the sphere formation assay that sevoflurane treatment ledto a significant decrease in the sphere formation ability of NSCs (Fig. 2B).

In addition, miR-18a is reported to inactivate several proliferation-related signaling pathways [8]. Therefore, we conjectured that miR-18a may affect the proliferation of NSCs. We constructed NSCs with miR-18a knockdown and overexpression, and the results showed that miR-18a was efficiently knocked down or inhibited (Fig. 2C).

To discern the effects of miR-18a on the proliferation of NSCs, we further detected the expression of Ki67 in cultured NSCs treated with sevoflurane after knockdown of miR-18a by immunofluorescence assay, and found that Ki67 expression was significantly decreased in sevoflurane treated NSCs and further transfection with miR-18a inhibitor significantly increased Ki67 expression (Fig. 2D). Meanwhile, the suppressed sphere-forming ability of NSCs induced by sevoflurane was found to be counterweighed by loss-of-function of miR-18a (Fig. 2E).

Furthermore, to study whether the development of the rat nervous system was mediated by sevoflurane via miR-18a, in vivo studies were also conducted. Neonatal rats (9-day-old) were then co-treated with sevoflurane and antagomiR-18a, the movement time of rats in the Morris water maze was measured, and the results showed that the movement time of rats treated with sevoflurane was significantly longer while additional treatment of antagomiR-18a significantly shortened the movement time (Fig. 2F). Additionally, the results of the open field experiment showed a similar trend: the movement distance of rats treated with sevoflurane was significantly longer while additional treatment of antagomiR-18a significantly shortened the movement distance (Fig. 2G). Conditioned fear test displayed that there was no significant difference regarding the freezing time to cue among groups, but the freezing time to context was significantly shorter in the sevoflurane-treated rats yet longer when antagomiR-18a was delivered (Fig. 2H, I).

Consistent with behavioral experiments, Western blot results showed that sevoflurane significantly inhibited the expression of neural cell marker proteins, NF-H/NF-M/NF-L, in both isolated cells and tissues, while inhibition of miR-18a expression on this basis restored the expression of neural cell marker proteins (Fig. 2J). Collectively, sevoflurane inhibits the proliferation of NSCs by upregulating miR-18a expression, thereby suppressing neural development in rats.

### miR-18a attenuates NSC proliferation and rat neurodevelopment by targeting RUNX1

**Fig. 3:**
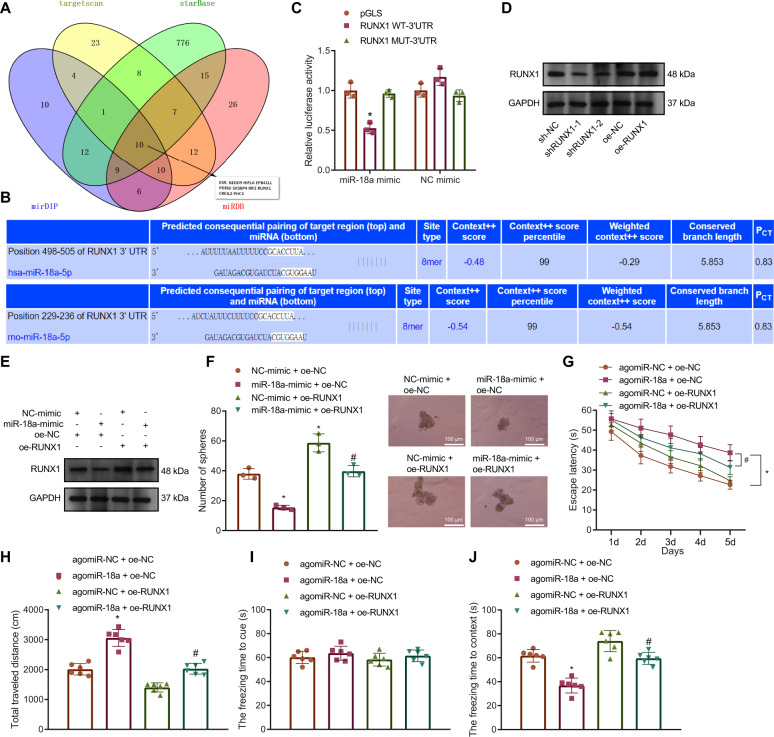
miR-18a downregulates RUNX1 expression to exert an effect on NSC proliferation and rat neurodevelopment. **A** Venn diagram displaying the intersection of miR-18a target genes predicted by starBase (http://starbase.sysu.EdUstaining.cn/), targetscan (http://www.targetscan.org/vert_72/), miRDB (http://mirdb.org/), mirDIP (http://ophid.utoronto.ca/mirDIP/) databases. **B** The binding sites between miR-18a and RUNX1 in human and rats detected by targetscan database. **C** The binding between miR-18a and RUNX1 detected by dual-luciferase reporter gene assay. **D** The expression of RUNX1 after shRUNX1-1, shRUNX1-2 or oe-RUNX1 treatment detected by Western blot analysis. **E** The expression of RUNX1 after miR-18a-mimic or oe-RUNX1 treatment detected by Western blot analysis. **F** Intuitive and statistical plots of the NSC sphere-forming ability after miR-18a-mimic or oe-RUNX1 treatment (bar = 100 μm). **G** The effect of RUNX1 expression on the movement time of rats in the water maze detected by Morris water maze assay. **H** The effect of RUNX1 expression on the movement distance of rats in the open field assessed by the open field test. **I** The effect of RUNX1 expression on the freezing time to cue of rats assessed by conditioned fear test. **J** The effect of RUNX1 expression on the freezing time to context of rats assessed by conditioned fear test. *p < 0.05 vs. the pGLS/NC-mimic + oe-NC/agomiR-NC + oe-NC group; ^#^*p* < 0.05 vs. the oe-NC/miR-18a-mimic + oe-NC. *n* = 6. The cell experiment was repeated 3 times independently.

To predict the downstream target genes of miR-18a, we searched starBase, targetscan, miRDB, and mirDIP databases and obtained 10 candidate target genes of miR-18a by intersection of the results (Fig. 3A). Previous literature revealed that RUNX1 was involved in the initial differentiation that regulates adult neurogenesis [10, 11]. Therefore, we speculated that RUNX1 may be a key downstream target gene of miR-18a in regulating NSC proliferation.

Further, we predicted a potential targeted binding site of miR-18a with RUNX1 by Targsetscan database (Fig. 3B). Validation by luciferase assay revealed that overexpression of miR-18a significantly inhibited wild-type (WT) RUNX1-3’-untranslated region (UTR) luciferase activity instead of its mutant type (MUT), indicating that miR-18a targets RUNX1 (Fig. 3C).

To verify the function of RUNX1, we constructed short hairpin RNAs (shRNAs) against RUNX1 (shRUNX1-1, shRUNX1-2), as well as its overexpression vector oe-RUNX1, and found by Western blot results that oe-RUNX1 significantly increased the protein expression level of RUNX1 while both shRUNX1-1 and shRUNX1-2 significantly inhibited the protein expression of RUNX1, of which shRUNX1-2 showed better silencing efficiency. So shRUNX1-2 was used for subsequent related experiments (Fig. 3D).

To interrogate the functional relationship between miR-18a and RUNX1, both miR-18a and RUNX1 were overexpressed in NSCs. First, it can be seen by Western blot that overexpression of miR-18a significantly reduced the protein level of RUNX1, while overexpression of miR-18a and RUNX1 restored the protein level of RUNX1 (Fig. 3E). Meanwhile, the detection on sphere-forming ability of NSCs showed that overexpressed miR-18a exerted inhibitory effect on sphere-forming ability while overexpression of RUNX1 led to opposite results. Moreover, overexpression of RUNX1 restored the inhibitory effect of overexpressed miR-18a on sphere-forming ability (Fig. 3F).

Neonatal rats (9-day-old) were injected with lentiviruses harboring agomiR-18a and/or RUNX1 at fixed points, respectively, and subjected to water maze test, open field test, and conditioned fear test to assess the effect of RUNX1 expression on NSCs in rat hippocampal tissues. The results unraveled that the movement time and distance of rats carrying agomiR-18a were significantly increased yet decreased after overexpressing miR-18 and RUNX1 together (Fig. 3G, H). Additionally, the results of the conditioned fear test indicated that there was no significant difference regarding the freezing time to cue of rats among groups, but the freezing time to context was significantly shorter in the rats overexpressing miR-18 alone while the rats overexpressing miR-18 and RUNX1 at the same time exhibited significantly longer the freezing time to context (Fig. 3I, J).

Altogether, miR-18a targets RUNX1 to inhibit NSC proliferation, ultimately suppressing rat neurodevelopment.

### RUNX1 promotes NSC proliferation by activating the Wnt/β-catenin signaling pathway

**Fig. 4:**
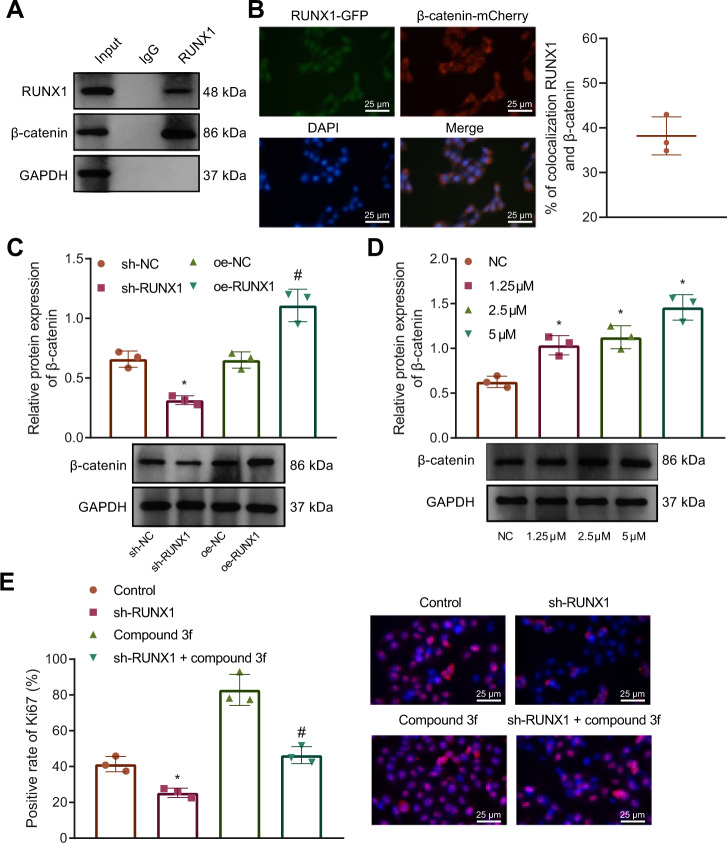
RUNX1 activates the Wnt/β-catenin signaling pathway to promote the proliferation of NSCs. **A** The binding of RUNX1 to β-catenin detected by Co-IP assay. **B** The co-localization of RUNX1 to β-catenin detected by immunofluorescence assay (bar = 25 μm); RUNX1-GFP: green fluorescence, β-catenin-mCherry: red fluorescence, DAPI: blue fluorescence. **C** The effect of RUNX1 overexpression/knockdown on the protein expression of β-catenin detected by Western blot analysis. **D** The protein expression of β-catenin under treatment of the Wnt/β-catenin signaling pathway activator compound 3 f at different concentrations detected by Western blot analysis. **E** The effect of RUNX1 overexpression/knockdown on the protein expression of proliferation factor Ki67 in NSCs detected by IHC staining (bar = 25 μm); PI: the nucleus in red fluorescence, Ki67: green fluorescence. *p < 0.05 vs. the sh-NC/NC/control group; ^#^*p* < 0.05 vs. the oe-NC/sh-RUNX1 group. The cell experiment was repeated 3 times independently.

The involvement of the Wnt/β-catenin signaling pathway in regulating the NSC proliferation and differentiation has been reported [13, 14]. To investigate whether RUNX1 affects neuronal cell proliferation by modulating the Wnt/β-catenin signaling pathway, we used a Co-immunoprecipitation (Co-IP) assay to validate whether RUNX1 interacts with β-catenin in normal NSCs, and the results confirmed the binding relationship between RUNX1 and β-catenin (Fig. 4A). In addition, immunofluorescence experiment revealed that RUNX1 co-localized with β-catenin, confirming the results of Co-IP (Fig. 4B). We also found that the expression of β-catenin was upregulated after oe-RUNX1 treatment, while sh-RUNX1 caused the decreased expression of β-catenin in NSCs as revealed by Western blot analysis (Fig. 4C).

Further, the Wnt/β-catenin signaling pathway activator compound 3 f was added in NSCs and β-catenin expression was significantly increased over the increase of compound 3 f concentrations (Fig. 4D). The proliferation of NSCs after addition of the Wnt/β-catenin signaling pathway activator compound 3 f in presence of RUNX1 knockdown was detected. Ki67 expression was significantly decreased after sh-RUNX1 treatment, accompanied by inhibited NSC proliferation. Also, the use of compound 3 f exerted inhibitory effect on NSC proliferation and offset the effect of RUNX1 reduction on NSC proliferation (Fig. 4E).

Taken together, RUNX1 promotes the proliferation of NSCs by activating the Wnt/β-catenin signaling pathway.

### Sevoflurane inhibits NSC proliferation by inducing miR-18a expression to block the RUNX1/Wnt/β-catenin signaling pathway

**Fig. 5:**
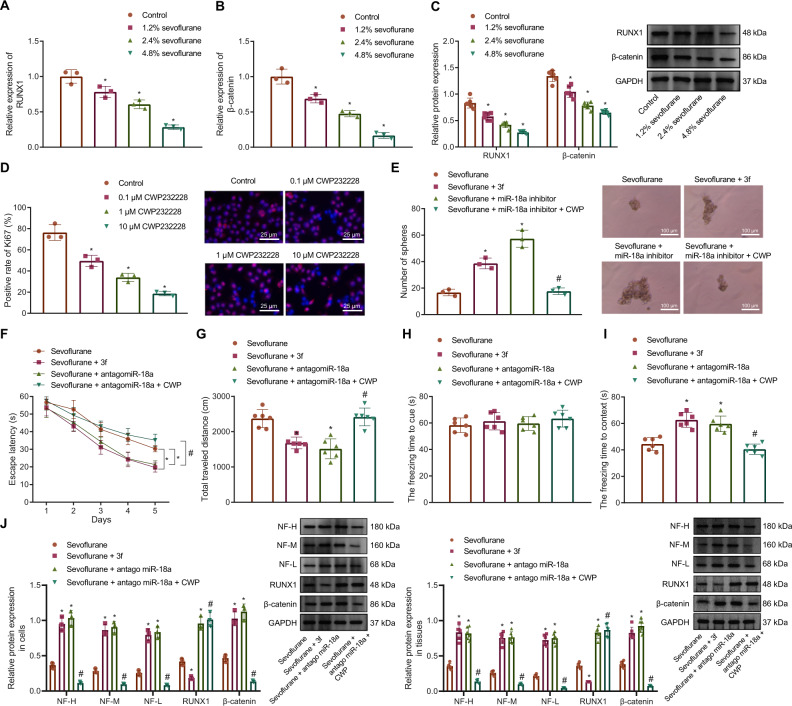
Sevoflurane regulates miR-18a-mediated RUNX1/Wnt/β-catenin signal pathway to suppress NSC proliferation and rat neurodevelopment. **A** The mRNA expression of RUNX1 under different concentrations of sevoflurane treatment detected by RT-qPCR. **B** The mRNA expression of β-catenin under different concentrations of sevoflurane treatment detected by RT-qPCR. **C** The protein expression of RUNX1 and β-catenin under different concentrations of sevoflurane treatment detected by Western blot analysis. **D** The expression of Ki67 protein in NSCs after different concentrations of CWP232228 treatment detected by IHC staining (bar = 25 μm); PI: the nucleus in red fluorescence, Ki67: green fluorescence. **E** Intuitive and statistical plots of sphere-forming ability of NSCs after sevoflurane, 3 f, miR-18a inhibitor, or CWP232228 treatment (bar = 100 μm). **F** The effect of sevoflurane, 3 f, miR-18a inhibitor, or CWP232228 treatment on the movement time of rats in the water maze assessed by mirris water maze test. **G** The effect of sevoflurane, 3 f, miR-18a inhibitor, or CWP232228 treatment on the movement distance of rats in the open field assessed by open field test. **H** The effect of sevoflurane, 3 f, miR-18a inhibitor, or CWP232228 treatment on the freezing time to cue of rats assessed by conditioned fear test. **I** The effect of sevoflurane, 3 f, miR-18a inhibitor, or CWP232228 treatment on the freezing time to context of rats assessed by conditioned fear test. **J** The expression of neural cell marker protein (NF-H/NF-M/NF-L) in cells and tissues detected by Western blot analysis. *p < 0.05 vs. the control/sevoflurane group; ^#^*p* < 0.05 vs. the sevoflurane + miR-18a inhibitor/sevoflurane + antagomiR-18a group. *n* = 6. The cell experiment was repeated 3 times independently.

It has been documented that sevoflurane inhibits the proliferation of NSCs by inhibiting β-catenin [15]. In order to investigate the regulatory effect of sevoflurane on the RUNX1/Wnt/β-catenin signaling pathway, we treated NSCs with different concentrations of sevoflurane and used untreated NSCs as control. RT-qPCR results showed that RUNX1 and β-catenin expression was downregulated in NSCs after sevoflurane treatment in a dose-dependent manner (Fig. 5A, B). In addition, Western blot results showed that RUNX1 and β-catenin protein expression showed a dose-dependent downregulation in the neonatal rat (9-day-old) hippocampal tissues after sevoflurane treatment (Fig. 5C).

For further verification on the action of the Wnt/β-catenin signaling pathway, NSC proliferation was detected after treatment using the inhibitor CWP232228 of different concentrations (0.1 μM, 1 μM and 10 μM) for 48 h. Immunohistochemistry (IHC) results revealed that Ki67 protein level was reduced over the increase of CWP232228 concentration, indicating that treatment with the Wnt/β-catenin signaling pathway inhibitor CWP232228 significantly inhibited NSC proliferation (Fig. 5D). In in vitro cell assays, miR-18a inhibitor and the Wnt/β-catenin signaling pathway inhibitor were used to treat sevoflurane-treated NSCs for detection on the resultant sphere-forming ability. It was found that miR-18a inhibitor or the Wnt/β-catenin signaling pathway activator promoted sphere-formation of the sevoflurane treated cells, while inhibiting Wnt/β-catenin signaling pathway activity significantly inhibited the contribution of miR-18a on sphere-forming ability of NSCs (Fig. 5E).

To further illustrate the relationship among the sevoflurane, miR-18a, and the Wnt/β-catenin signaling pathway, water maze test, open field test, and conditioned fear test were performed. The results showed that under sevoflurane treatment, the movement time in the water maze and movement distance in the open field were significantly reduced in rats injected with the Wnt/β-catenin signaling pathway activator compound 3 f, which was similar to that in rats received treatment of sevoflurane and antagomiR-18, while opposite results were observed in response to treatment of sevoflurane + antagomiR-18 + CWP232228 when compared with treatment of sevoflurane + antagomiR-18 (Fig. 5F, G). The results of the conditioned fear test showed that there was no significant difference in the freezing time to cue among the rats, but the freezing time to context time in the rats injected with the Wnt/β-catenin signaling pathway activator compound 3 f or antagomiR-18 was significantly longer than that in the rats injected with sevoflurane alone, and the freezing time to context was shortened after the use of the Wnt/β-catenin signaling pathway inhibitor CWP232228 in presence of sevoflurane + antagomiR-18a (Fig. 5H, I).

Similar to the behavioral experiments, Western blot results showed that after treatment with sevoflurane, addition of the Wnt/β-catenin signaling activator compound 3 f or antagomiR-18 significantly increased β-catenin/NF-H/NF-M/NF-L protein levels, but the Wnt/β-catenin signaling activator compound 3 f inhibited RUNX1 expression while antagomiR-18 upregulated RUNX1 expression under sevoflurane treatment; moreover, Wnt/β-catenin signaling pathway inhibitor CWP232228 significantly downregulated β-catenin/NF-H/NF-M/NF-L protein levels when sevoflurane + antagomiR-18 were present (Fig. 5J).

In a word, sevoflurane can inhibit NSC proliferation by inducing miR-18a expression and inhibiting the RUNX1/Wnt/β-catenin signaling pathway activation, ultimately inhibiting rat neurodevelopment.

## Discussion

**Fig. 6:**
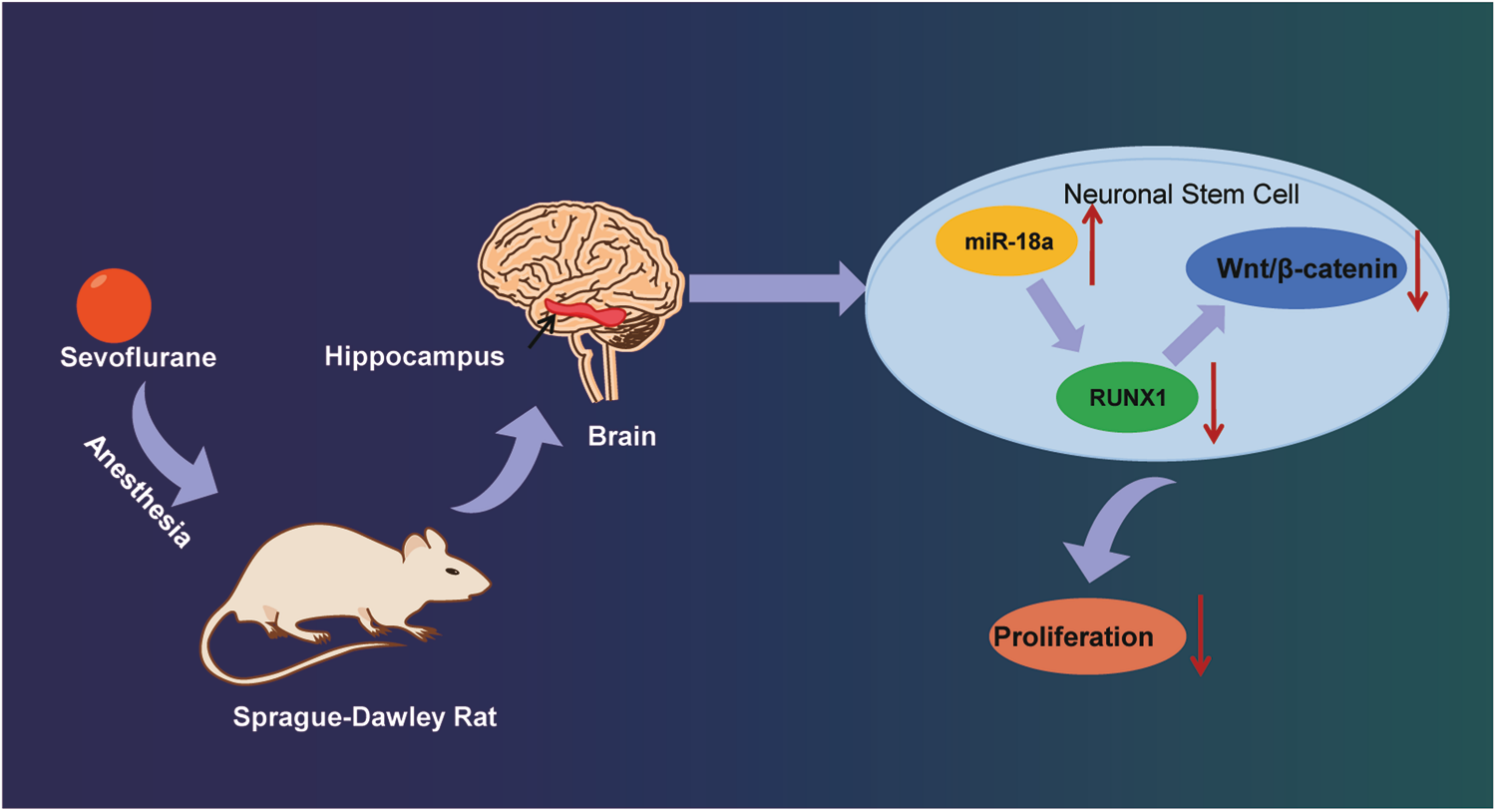
Schematic diagram of the molecular mechanism by which sevoflurane inactivated the RUNX1/Wnt/β-catenin signaling pathway through miR-18a upregulation to regulate NSC proliferation in 9-day-old neonatal rats. Sevoflurane inactivates the RUNX1/Wnt/β-catenin signaling pathway by inducing miR-18a expression, whereby inhibiting NSC proliferation and affecting rat neurodevelopment.

Numerous studies have reported that embryonic or neonatal exposure to sevoflurane inhibits NSC proliferation in rat hippocampal tissues and impairs learning and memory ability in rats [3, 16–19]. Hence, the safety of sevoflurane and other anesthetic agents has been questioned. In our present study, we draw a preliminary conclusion that sevoflurane inactivates the RUNX1/Wnt/β-catenin signaling pathway by inducing miR-18a expression, whereby inhibiting NSC proliferation and affecting rat neurodevelopment (Fig. 6).

In the initial stage, we found that sevoflurane upregulated the expression of miR-18a in rat hippocampal tissues and NSCs. The contribution of sevoflurane to the noticeable changes in the expression of miRNAs has been demonstrated. A recent example is that sevoflurane plays an inhibitory role in the proliferation and metastasis of glioma cells by upregulating miR-124-3p [20]. Dysregulated hippocampal miRNAs have also been implicated in the function of sevoflurane in impeding neural development [21]. For instance, miR-124 promotes proliferation and neural differentiation of NSCs by inhibiting the expression of dishevelled binding antagonist of beta-catenin 1 and activating the Wnt/β-catenin pathway [22]. Also, miR-374b is reported as a promising target for adjusting NSC-modulated neurogenesis by targeting hairy and enhancer of split 1 [23]. Likewise, Li et al. have pointed out that miR-18a regulates neural precursor cells and fulfills a pivotal role in the cell proliferation [8], and miR-18a is indicated to decrease the percentage of the proliferative factor Ki67-postive cells [24], which is partially consistent with our study.

Next, our report found that miR-18a targeted RUNX1 to limit the proliferation of NSCs. It has been reported that RUNX genes serve as vital regulators for the transition between proliferation and differentiation, and regulate the stem cell proliferation and differentiation in multiple cell types including NSCs [10]. RUNX1, a member of the RUNX family, controls the growth and differentiation of certain NSCs and has the ability to promote NSC differentiation to repair the injured complex nervous system [11]. Additionally, the transcription factor RUNX1 was reported to be a target gene of miR-18a-5p [25], and RUNX1was directly targeted by miR-18a in gastric cancer cells [26]. Given the above evidence, the inhibitory role of miR-18a in RUNX1 expression and the miR-18a/RUNX1 axis functioning in NSC proliferation can be verified.

Furthermore, our findings uncovered the potential of RUNX1 to promote NSC proliferation by activating the Wnt/β-catenin signaling pathway. Wnt signaling is considered as a balance regulator between the proliferation and differentiation of NSCs through the β-catenin, the transcriptional coactivator of Wnt, in the process of tissue homeostasis and neuronal development, and disrupting the Wnt signaling leads to neurological diseases as well as developmental defects [14]. Another report has also highlighted the critical involvement of the Wnt/β-catenin signaling pathway in stem cell maintenance and embryonic development [27]. Of note, Sweeney *et al*. have highlighted the complex interplay between the RUNX genes and the Wnt/β-catenin signaling pathway [28]. What’s more, RUNX1 contributes to activating the Wnt/β-catenin signaling pathway and the epithelial to mesenchymal transition in colorectal cancer [12]. Collectively, RUNX1 facilitates NSC proliferation by promoting activation the Wnt/β-catenin signaling pathway. Also, our behavioral experiments showed that RUNX1 overexpression protected rat neurodevelopment, while sevoflurane resulted in nerve injury in rats. Consistently, neonatal exposure to sevoflurane was found to induce aberrant social behaviors in mice under fear conditioning [29]. In addition, sevoflurane has been indicated to attenuate the proliferation and differentiation of NSCs by inhibiting the Wnt/β-catenin signaling pathway [15], further confirming the role of the sevoflurane-mediated Wnt/β-catenin signaling pathway in neurodevelopment. Altogether, it is validated that sevoflurane suppresses NSC proliferation and impairs rat neurodevelopment via the miR-18a-regulated RUNX1/Wnt/β-catenin signaling pathway.

To sum up, this paper proposes a new molecular mechanism by which sevoflurane and miR-18a inactivated the RUNX1/Wnt/β-catenin signaling pathway to impair neural development. We hope to provide a new theoretical basis for understanding the mechanism of sevoflurane-induced neurotoxicity and aid in choosing more reasonable anesthetics for patients to ensure their safety. Also, whether this phenomenon is acting only on NSC population remains unclear with further work required to deepen understanding.

## Materials and methods

### Ethics statement

Animal experimentations were ratified by the Animal Ethics Committee of The First Center of Chinese PLA General Hospital and Hainan Hospital of Chinese PLA General Hospital and completed in accordance with the *Animal Welfare Law*. Due efforts were made to reduce animal pain.

### Bioinformatics analysis

Microarray dataset GSE141242 of rat hippocampal tissues after sevoflurane anesthesia was obtained from the GEO database, including 3 normal control rats and 3 sevoflurane-treated rats. Its differential analysis was completed utilizing R language the “limma” package with the threshold being∣logFoldChange∣ > 0.3 and *p* < 0.05. Then, starBase, mirDIP, miRDB and targetscan were employed to predict downstream target genes of miRNAs.

### Experimental animals and grouping

Neonatal rats (9-day-old) were procured from Shanghai Laboratory Animal Co., Ltd. (Shanghai, China), and randomized with a female to male ratio of 1:1. During behavioral experiments such as water maze learning and conditioned fear test, rates were subjected to the corresponding training, then anesthetized with different contents of sevoflurane and awakened 6 h later for various experiments (*n* = 6). In addition, rats were treated with sevoflurane of varying concentrations, and their hippocampal tissues were isolated after 6 h anesthesia and cryopreserved.

### Lentiviral infection

Lentiviral packaging plasmids for sh-RUNX1, oe-RUNX1, as well as negative control (NC) were developed by Shanghai GenePharma Co., Ltd. (Shanghai, China), and at the same time, agomiR-18a, agomiR-NC, antagomiR-NC, and antagomiR-18a were prepared. Six rats in each group were anesthetized with isoflurane (gas) and fixed on a rat brain stereotaxic instrument (Stoelting). A low-voltage DC insulating pad was applied during the operation for heat preservation. The head was shaved and disinfected with iodine and alcohol, the skin was incised 1–2 cm in a sagittal position along the middle of the head, and the skull was fully exposed to determine the coordinates of the rat hippocampal tissue area (AP = −3.5 mm; DV = −2.8 mm; ML = −2 mm).

Afterwards, a hole was drilled at the coordinate site using a cranial drill and 5 μL lentivirus (2 × 10^9^ TU/mL) or 4 μL agomiR-18a or antagomiR-18a (both 0.8 nmol) was injected with a microsyringe according to the coordinates. The wound was sutured postoperatively with application of iodophor, and the rats were placed in a rat cage after awaking. Two weeks after the injection, the rats were subjected to various behavioral tests, and then, all rats were anesthetized by isoflurane and sacrificed by cervical dislocation, followed by separation of hippocampal tissues for RT-qPCR and immunoblotting.

### Water maze and open field tests

For measuring the acquisition of learning behavior in rats, the rats were subjected to a navigation test for 5 consecutive days. After training, each rat was treated with sevoflurane at different percentages (1%, 2%, or 3%), and awakened 6 h later for a spatial exploration test. The total distance traveled while swimming and the distance swum 1 min in the target quadrant were recorded by the tracking system after removing the platform [30].

The open field test was used to assess movement, exploration and anxiety-like behavior in rats utilizing a square area (100 × 100 × 40 cm) in the open field [31].

### Conditioned fear test

Rats were placed in the animal housing room for one-week adaptation with adjusted temperature and light: dark period of 12 h: 12 h. They were randomly divided into cages and housed separately and given free access to food and water. Conditioned fear training was performed according to previous studies [32, 33], and then the freezing time to context and the freezing time to cue were recorded.

### RT-qPCR

Total RNA from NSCs and hippocampal tissue samples was extracted employing Trizol reagent (Invitrogen, Carlsbad, CA), and miRNAs from cells and hippocampal tissue samples were extracted utilizing miRNA extraction kit (19331ES08, MolPure ® Cell/Tissue miRNA Kit, Yeasen, Shanghai, China). The extracted RNA was reversely transcribed into complementary DNA (cDNA) from 500 ng of RNA by referring to the PrimeScript RT reagent Kit (RR047A, Takara, Japan). miRNA was reversely transcribed into cDNA utilizing microRNA Reverse Transcription Kit (EZBioscience, EZB-Exo-RN1). The synthesized cDNA was subjected to RT-qPCR with the Fast SYBR Green PCR kit (4364344, Applied biosystems, Foster City, CA) and the ABI PRISM7300 RT-qPCR system (Applied biosystems). GAPDH served as a normalizer for mRNA, and U6 for miRNA. The relative expression of genes or mRNAs was analyzed employing the 2^-ΔΔCt^ method. Primers are described in Table S1.

### IHC

Fresh rat hippocampal tissue samples were fixed, embedded in paraffin, sectioned, deparaffinized, dehydrated with alcohol of gradient concentrations, and treated with 3% methanol H_2_O_2_ for 20 min with 0.1 M PBS for 3 min. After antigen retrieval, the tissue sections were sealed with normal goat serum blocking solution (C-0005, Shanghai Haoran Biotechnology Co., Ltd., Shanghai, China) at ambient temperature for 20 min and probed with primary antibody (rabbit antibody to Ki67, ab16667, 1: 500, Abcam, Cambridge, UK; β-catenin, ab32572, 1: 200, Abcam; RUNX1, ab92336, 1: 200, Abcam) overnight at 4 °C. After that, the sections were re-probed with corresponding fluorescence-labeled secondary antibody and sealed with neutral resin. Finally, 5 randomly selected high-power fields from the sections were observed and photographed under the microscope, with 100 cells counted in each field. To verify the validity of RUNX1 antibody, a control group was set by incubating the PBS-treated tissue sections with corresponding fluorescence-labeled secondary antibody while no fluorescence signal was detected.

### Isolation, culture, and identification of NSCs

Two neonatal rats (aged 9 d) were weighed and sacrificed, and the brain tissues were isolated under sterile conditions and then rinsed thoroughly in D-Hanks solution. The meninges and superficial vessels were dissected under a dissecting microscope, and the hippocampal tissues were precisely dissected, cut into pieces with an ophthalmic scissor, and then incubated in Dulbecco’s modified Eagle’s medium (DMEM) (11569077, Gibco, Carlsbad, CA): Nutrient mixture F-12 (DMEM/F-12) medium (11330-032, Gibco) replenishing 10% fetal bovine serum (10099, Gibco), and 0.1% penicillin-streptomycin (15140-148, Gibco). The hippocampal tissues were prepared into single-cell suspension, filtered through 200 meshes and 400 meshes, and centrifuged at 157 × *g* for 5 min with the supernatant discarded. The cells were resuspended in basal medium and the cell density was adjusted to 5 × 10^5^ cells/mL, and then the cell suspension was seeded in a 25 cm^2^ culture flask and cultured in a 5% CO_2_ incubator at 37 °C, with the medium renewed every 2 to 3 d. Following 5–7 d incubation, the cells were transferred to new culture flasks in the light of the conditions and density. NSC clone spheres on the 0, 3rd and 14th day during primary culture were dropped onto a coverslip pre-coated with polylysine and cultured with a small amount of culture medium for another 12 h to ensure that the clone spheres were completely attached to the coverslip without differentiation. After 12 h, the plates were removed and immunofluorescent staining was processed to quantify nestin protein (ab221660, 1: 200, Abcam).

### Isolation, culture and identification of astrocytes and oligodendrocytes

Two neonatal rats (aged 9 d) were weighed and sacrificed, and the brain tissues were isolated under sterile conditions and then rinsed thoroughly in D-Hanks solution. Rat cerebral cortex was dissected under a dissecting microscope, from which astrocytes were isolated and preserved in Hanks’ HEPES buffer. Then, astrocytes were filtered using a 100-mesh strainer, centrifuged at 1000 g for 15 min, then resuspended in DMEM + 10% FBS, and cultured in a dish plated with polylysine. Following fixation using 4% paraformaldehyde, astrocytes were treated with 0.5% Triton X-100 at 4 °C, incubated with GFAP (ab7260, 1:200, Abcam, Cambridge, UK) overnight, and treated with a fluorescent secondary antibody (1:400 dilution) for 1 h, followed by observation under a fluorescence microscope.

After sacrifice, the hindbrain (cerebellum and brainstem) of rats was separated from the forebrain under a dissecting microscope. Then, a Petri dish with the separated forebrain was placed under a dissociating microscope, the two hemispheres were separated along the midline, and the meninges were pulled out of the brain tissue with the meninges and blood vessels removed. Fine forceps or a 1 ml pipette tip was used to dissociate the tissue in a rough manner, and then the MACS neural dissociation kit (130-092-628, Miltenyi, San Diego, CA, USA) was used to isolate oligodendrocytes. Briefly, cells were resuspended in prewarmed DMEM medium after tissue digestion with the enzymes in the kit, and then filtered through 70 μm and 40 μm filters. Oligodendrocytes were isolated using MACS O4 + magnetic beads according to the kit instructions, and then cultured in poly-lysine-coated petri dishes. Oligodendrocytes were fixed with 4% paraformaldehyde, treated with 0.5% Triton X-100 at 4 °C, incubated with oligodendrocyte marker protein O4 (MAB1326, 1:200, R&D Systems, Minnesota, USA) overnight, with a fluorescent secondary antibody (1:400 dilution) for 1 h, and finally observed under a fluorescence microscope.

### Transfection of NSCs

NSCs at the logarithmic phase were detached with trypsin, seeded in 6-well plates at 1 × 10^5^ cells per well and routinely cultured for 24 h. After achieving about 75% confluence, cell transfection was started employing Lipofectamine 2000 (11668019, Invitrogen), and after plasmid transfection for 48 h, the transfection efficiency of sh-RUNX1, oe-RUNX1, miR-18a mimic, and miR-18a inhibitor was quantified utilizing RT-qPCR. The above plasmids were procured from Shanghai GenePharma, and were used at a concentration of 50 ng/mL. The cells were treated with DMSO, CWP232228 (Wnt/β-catenin signaling pathway inhibitor, 0.1, 1, or 10 μM, HY-18959, MedChemExpress, NJ), or compound 3 f (Wnt/β-catenin signaling pathway activator, 1.25, 2.5, and 5, HY-114321, MedChemExpress). The RUNX1 silencing sequences are depicted in Table S2.

### Sevoflurane treatment of NSCs, astrocytes, and oligodendrocytes

Neonatal NSCs, astrocytes, and oligodendrocytes at passage 3–4 were suspended in DMEM and seeded into 6-well plate (cell concentration of 1 × 10^6^ cells/mL). After inoculation, the culture medium was placed in a perspex box under constant moist and sealed conditions, and the anesthetic gas was injected into the chamber with the help of a graduated evaporator (Drager, Lübeck, Germany) which contained a mixture of 95% air and 5% CO_2_ at a rate of 0.5 L/min. The concentration of anesthetic gas was monitored at the entrance of the closed chamber using a gas monitor (PM8050; Drager), and the scale of the evaporator was adjusted to maintain the sevoflurane concentration at 4.8%. The control group was placed in the incubator under the same conditions, and added with a mixture of 95% air and 5% CO_2_ but with no anesthetic gas. The exposure time of the cells in the above gases was 6 h.

### Immunoblotting

Cultured cells were detached by trypsin, collected and lysed with enhanced RIPA lysis containing protease inhibitors (Wuhan Boster Biological Technology Co., Ltd., Wuhan, Hubei, China) with the protein concentration measured using BCA protein quantification kit (Boster). Proteins were separated by 10% SDS-PAGE, and the separated proteins were electrotransferred onto a polyvinylidene fluoride membrane and blocked with 5% bovine serum albumin (BSA) for 2 h at ambient temperature to block non-specific binding. The membrane was incubated with diluted primary antibodies (rabbit anti-GAPDH, ab9485, 1: 500, Abcam; rabbit anti-RUNX1, ab272456, 1: 500, Abcam; NF-H, 1: 500, #55453, Cell Signaling Technology [CST], Danvers, MA; NF-M, 1: 500, #84390, CST; NF-L, 1: 500, ab223343, Abcam; β-catenin, 1: 500, ab32572, Abcam) overnight at 4 °C. Then the membrane was then incubated with horseradish peroxidase-labeled goat anti-rabbit secondary antibodies (ab205719; 1: 2000; Abcam) for 1 h, treated with enhanced chemiluminescence working fluid (WBULS0100, EMD Millipore, Billerica, MA) and placed in an optical luminescent instrument (AS4000, GE, Little Chalfont, Buckinghamshire, UK) to develop images. Finally, Image J software was run to quantify the grayscale of each band in the Western blot image normalized to GAPDH.

### Dual-luciferase reporter gene assay

The downstream target genes of miR-18a were predicted using a biological prediction website, and the results indicated RUNX1 as a direct target gene of miR-18a, which was further validated by luciferase assay. The cDNA fragment of RUNX1 3’-UTR with miR-18a binding site (WT-RUNX1-3’-UTR: 5’-AUUUUUUAAUUUUUCCGCACCUUA-3’) was inserted into the pmirGLO vector. Another cDNA fragment of RUNX1 3’-UTR with binding site mutation (Mut-RUNX1-3’-UTR: 5’-AUUUUUUAAUUUUUUCCCGUAAUCGA-3’) was constructed using site-directed mutagenesis and inserted into the pmirGLO vector. Next, the correctly sequenced pmirGLO-RUNX1 or pmirGLO-mutRUNX1 recombinant vectors were transfected into HEK293 cells (CRL-1573, American Type Culture Collection, Manassas, VA) by lipofection with miR-18a mimic or NC. After 48 h of culture, the activities of Renilla luciferase and firefly luciferase were detected using a multimodal reader (SpectraMax M5, Molecular Devices, Sunnyvale, CA) with a set time interval of 2 s and an assay time of 10 s.

### EdU staining

A total of 1 × 10^5^ cells were seeded in a 24-well plate, and after 24 h of cell culture, EdU was added to the culture medium to reach a concentration of 10 µmol/L. Following 48-h of incubation, the medium was aspirated, the cells were fixed with methanol solution for 15 min at ambient temperature, washed twice with PBS containing 3% BSA, and incubated with PBS containing 0.5% Triton-100 for 20 min at ambient temperature. Then, the cells were incubated with freshly prepared Click-iT reaction mixture containing azide-conjugated Alexa594 (A-11037, Invitrogen) for 30 min at ambient temperature in the dark, and DAPI (D9542, Sigma-Aldrich, St Louis, MO) was added to stain the nucleus for 5 min, after which stained cells were examined with a NikonEclipse E600 fluorescence microscope, and photographed with a Retiga1300Q imaging camera. To determine the percentage of EdU-stained positive cells, the number of cells with red fluorescence (Alexa594-stained) was divided by the number of cells with blue fluorescence (DAPI-stained).

### Sphere formation assay

The self-renewal capacity of NSCs was assessed using a neurosphere formation assay. The isolated primary NSCs were seeded in 24-well plates at a density of 200 cells/well, treated with sevoflurane or transfected 24 h later. After culture for 7 d, the culture medium was discarded, and the cells were carefully washed 3 times with sterile PBS followed by the addition of 300 µL 1% crystal violet solution to stain the neurospheres. Under a microscope, colonies larger than 20 μm in diameter were counted and the colony formation rate was calculated.

### Co-IP

A total of 600 μL RIPA buffer (P0013C, Shanghai Beyotime Biotechnology Co., Ltd., Shanghai, China) containing protease inhibitor was added to the cells to completely lyse the cells, then the cells on ice were scraped and the supernatant was harvested by centrifugation. Supernatants of cell lysates were incubated with the indicated antibodies RUNX1 (ab272456, Abcam) or IgG (ab182931, Abcam) or Protein A/G PLUS-agarose beads (IP10, Sigma-Aldrich) for 12 h at 4 °C. After IP, the beads were washed thoroughly with cell lysis buffer. Finally, 60 μL immunoprecipitated protein and 1 × SDS PAGE were boiled for 10 min, and the precipitated protein was then analyzed by immunoblotting.

### Statistical analysis

All data were processed by SPSS 21.0 statistical software (IBM, Armonk, NY). Measurement data were expressed as mean ± standard deviation. Unpaired *t* test was adopted for comparison between two groups, and one-way analysis of variance (ANOVA) for comparison among multiple groups. Data comparison among multiple groups at different points was conducted using repeated measures ANOVA and Tukey’s post hoc test. A value of *p* < 0.05 described a significant difference.

## Supplementary information

Supplementary files

original western blots

## Data Availability

The datasets generated/analysed during the current study are available at the manuscript and supplemental files.
