# Supplementary material for: Sevoflurane induces microRNA-18a to delay rat neurodevelopment via suppression of the RUNX1/Wnt/β-catenin axis
Source: Cell Death Discov. 2022 Oct 1;8:404. doi: 10.1038/s41420-022-01179-y (PMC9526732; doi:10.1038/s41420-022-01179-y)
Supplement: Supplementary file 1 — Supplementary files [file 41420_2022_1179_MOESM1_ESM.docx]

**Table S1** Primer sequences for RT-qPCR

| Gene | Sequence |
| --- | --- |
| miR-18a | F: 5’-TAAGGTGCATCTAGTGCAGATAG-3’ |
|  | R: Universal reverse primer |
| U6 | F: 5’-CTCGCTTCGGCAGCACATA-3’ |
|  | R: Universal reverse primer |
| RUNX1 | F: 5’-TGAGCTGAGAAATGCTACCGC-3’ |
|  | R: 5’-ACTTCGACCGACAAACCTGAG-3’ |
| β-catenin | F: 5’-ATCATTCTGGCCAGTGGTGG-3’ |
|  | R: 5’-GACAGCACCTTCAGCACTCT-3’ |
| GAPDH | F: 5’-AGACAGCCGCATCTTCTTGT-3’ |
|  | R: 5’-TACGGCCAAATCCGTTCACA-3’ |

Note: RT-qPCR, reverse transcription quantitative polymerase chain reaction; miR, microRNA; RUNX1, runt related transcription factor 1; GAPDH, Glyceraldehyde-3-phosphate dehydrogenase; F: Forward; R: Reverse.

**Table S2** shRNA sequences for RUNX1

| Target | Sequences |
| --- | --- |
| shRUNX1-1 | CCGGTTCGTACCCACAGTGCTTCATCTCGAGATGAAGCACTGTGGGTACGAATTTTTG |
| shRUNX1-2 | CCGGCTACGATCAGTCCTACCAATACTCGAGTATTGGTAGGACTGATCGTAGTTTTTG |

Note: shRNA, short hairpin RNA; RUNX1, runt related transcription factor 1.


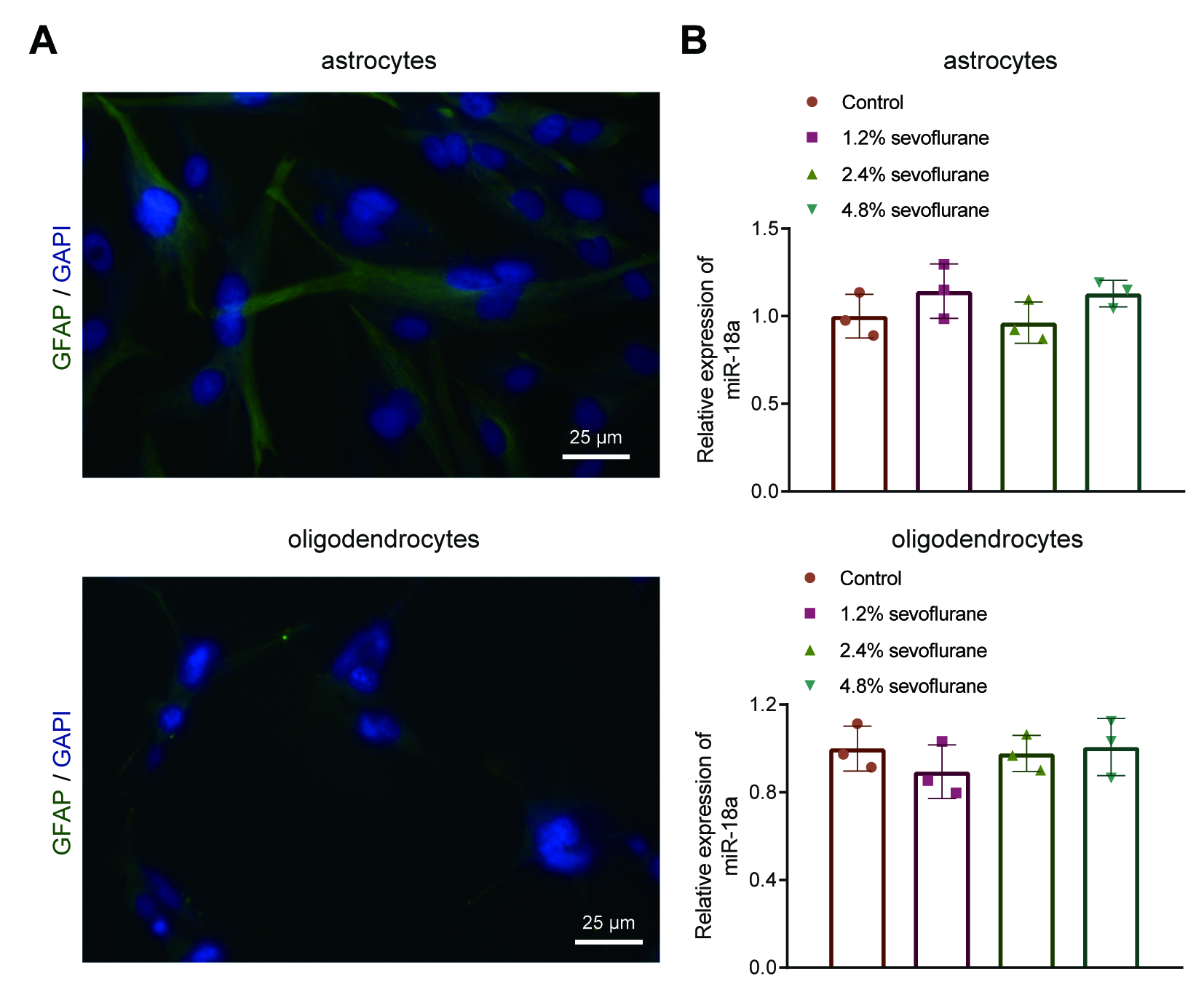


**Supplementary Fig. 1** Identification of astrocytes and oligodendrocytes and detection of miR-18a expression changes. A, Expression of astrocyte and oligodendrocyte positive marker proteins GFAP (green) and O4 (green) under fluorescence microscope, DAPI: nucleus, blue fluorescence (bar = 25 μm). B, The expression of miR-18a in astrocytes and oligodendrocytes was detected by RT-qPCR under different concentrations of sevoflurane.
