# Supplementary figures and images for: Sevoflurane induces microRNA-18a to delay rat neurodevelopment via suppression of the RUNX1/Wnt/β-catenin axis
Source: Cell Death Discov. 2022 Oct 1;8:404. doi: 10.1038/s41420-022-01179-y (PMC9526732; doi:10.1038/s41420-022-01179-y)

Figure 2J


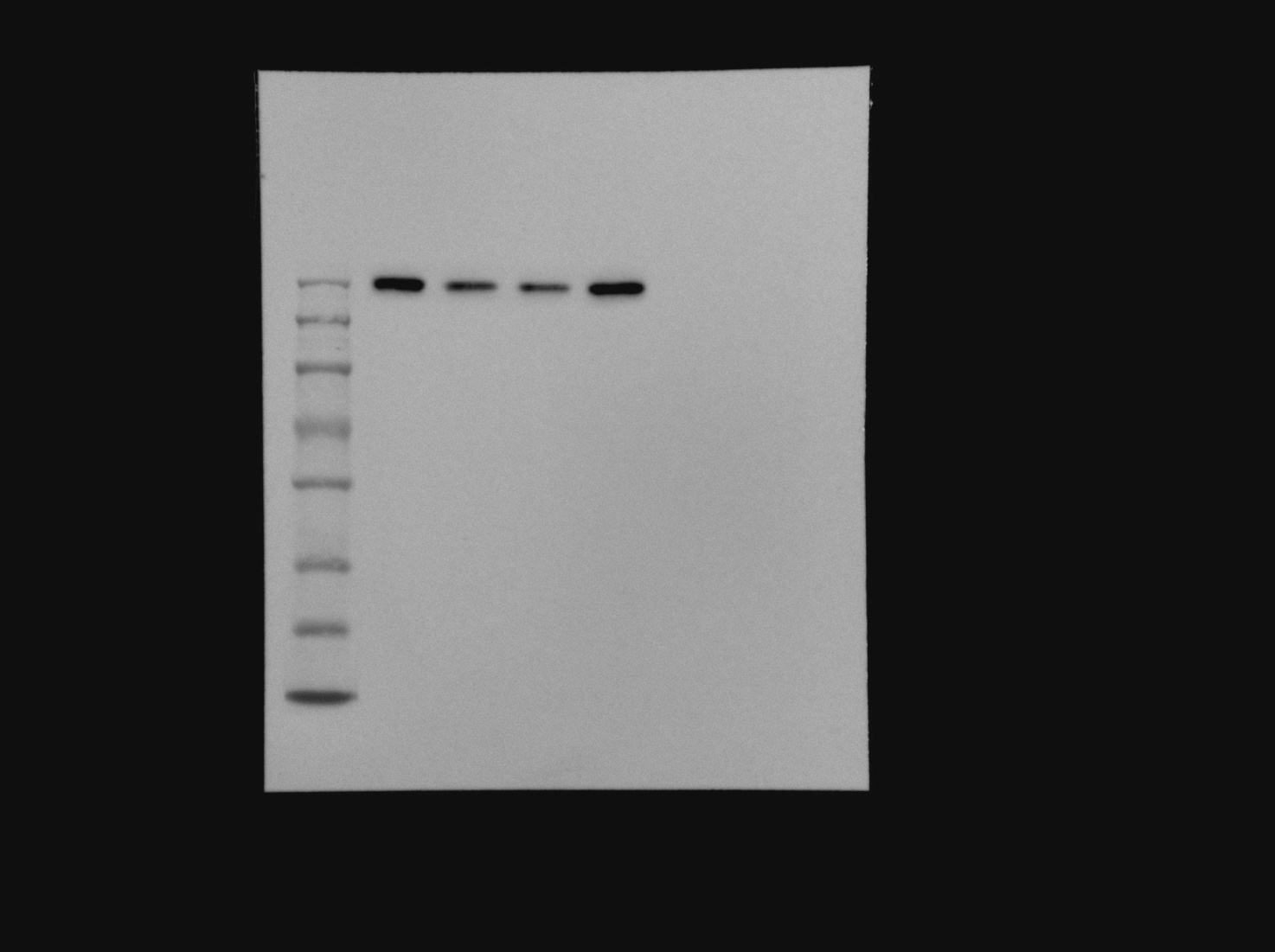


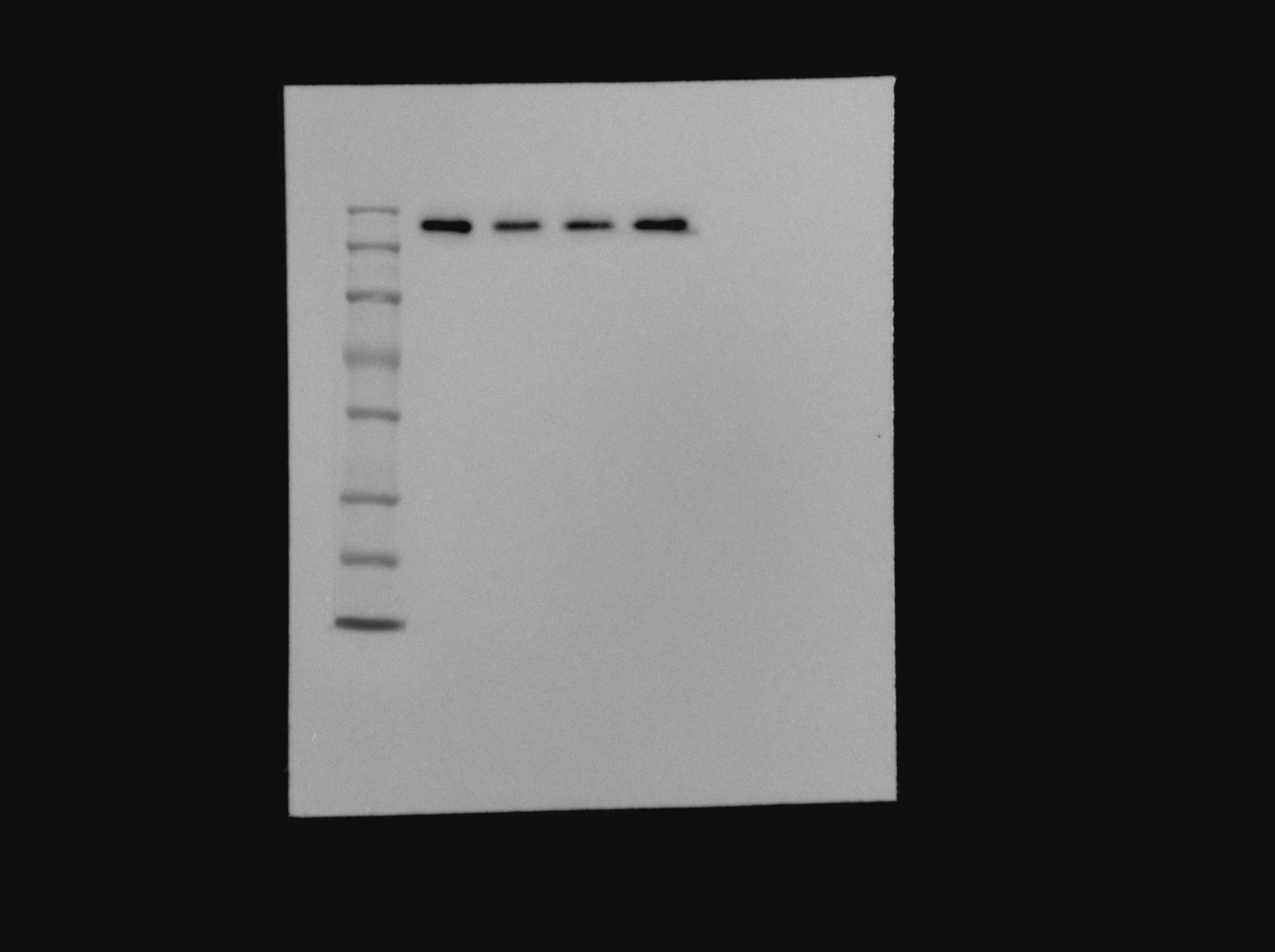


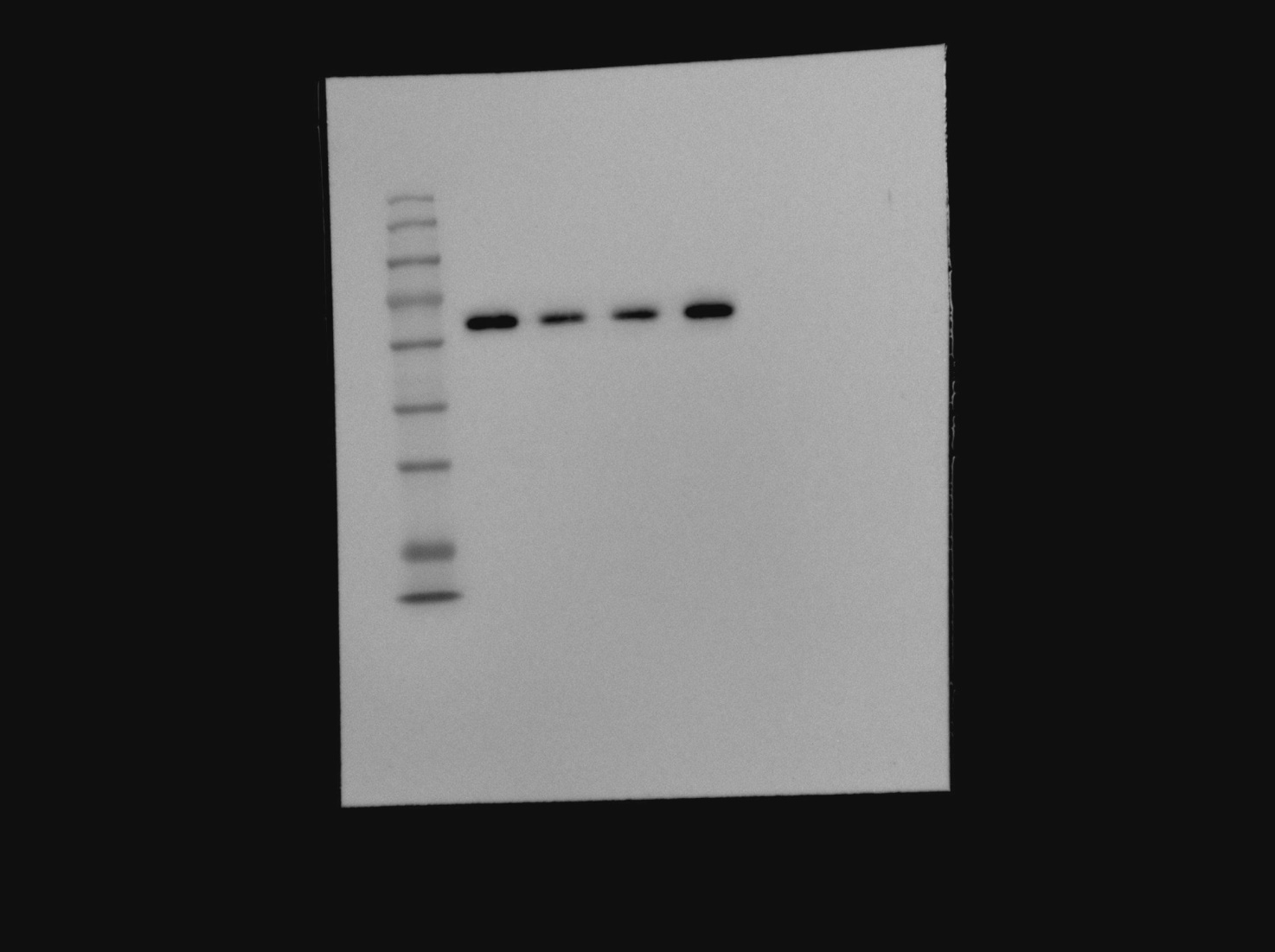


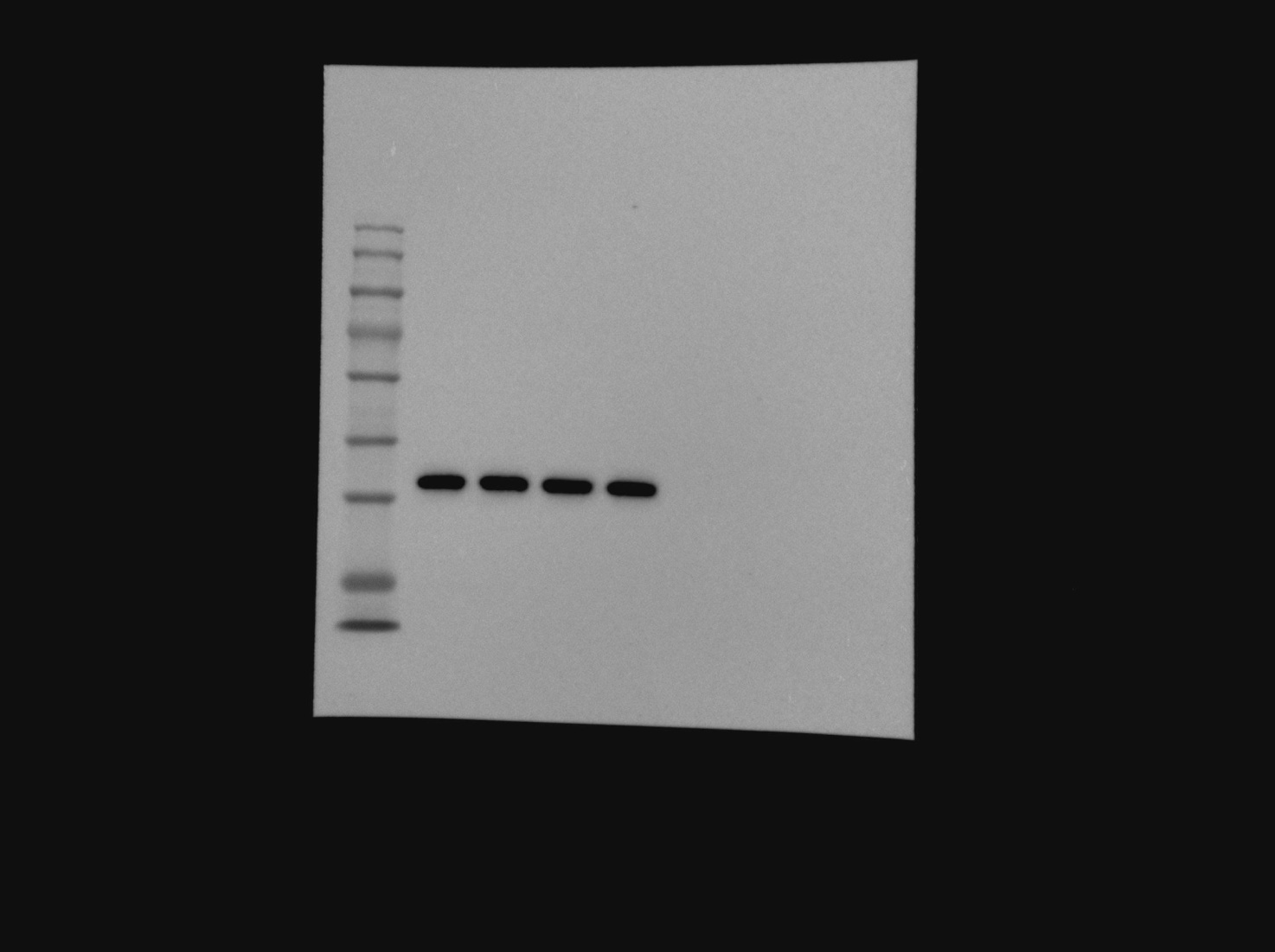


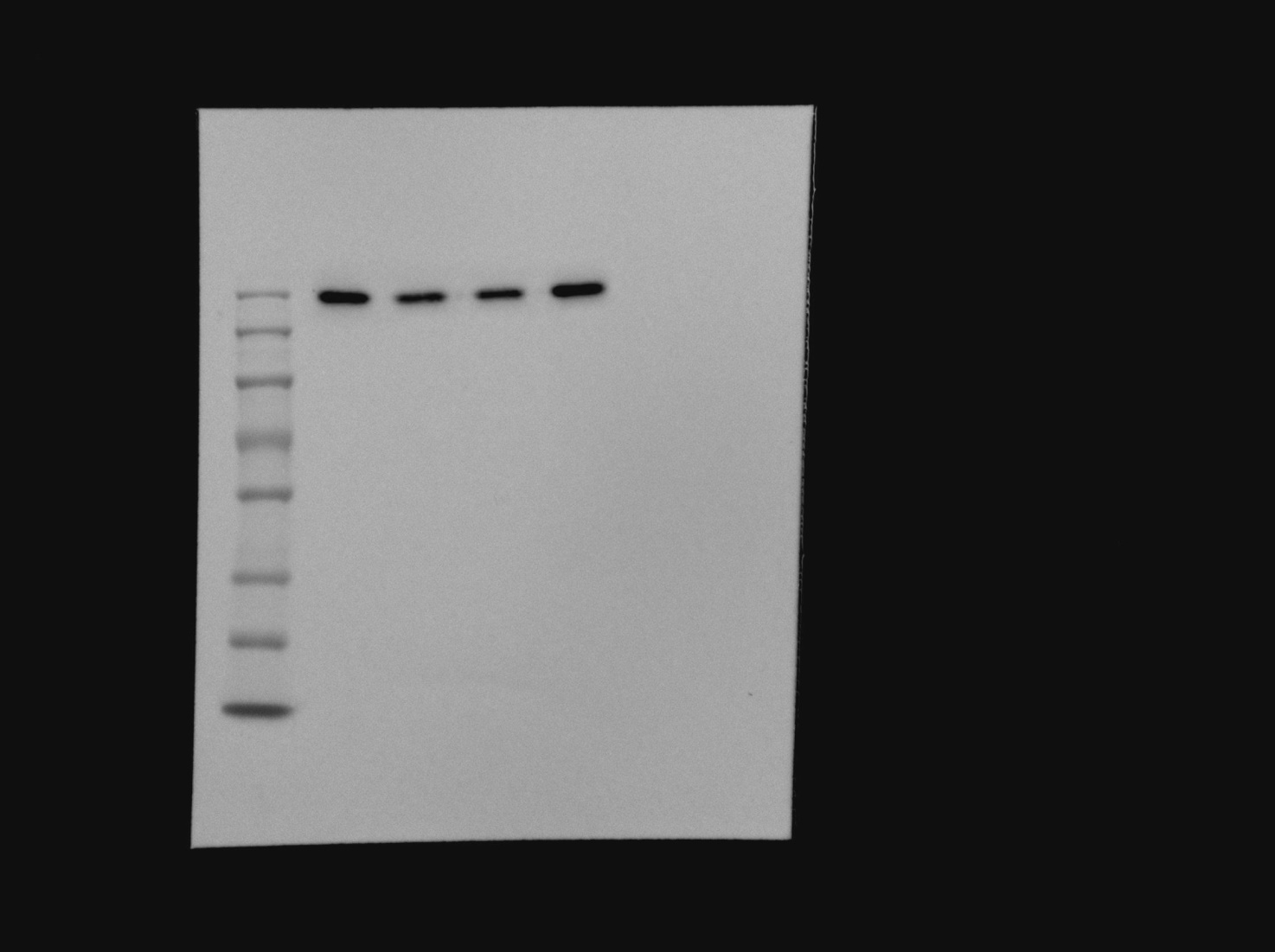


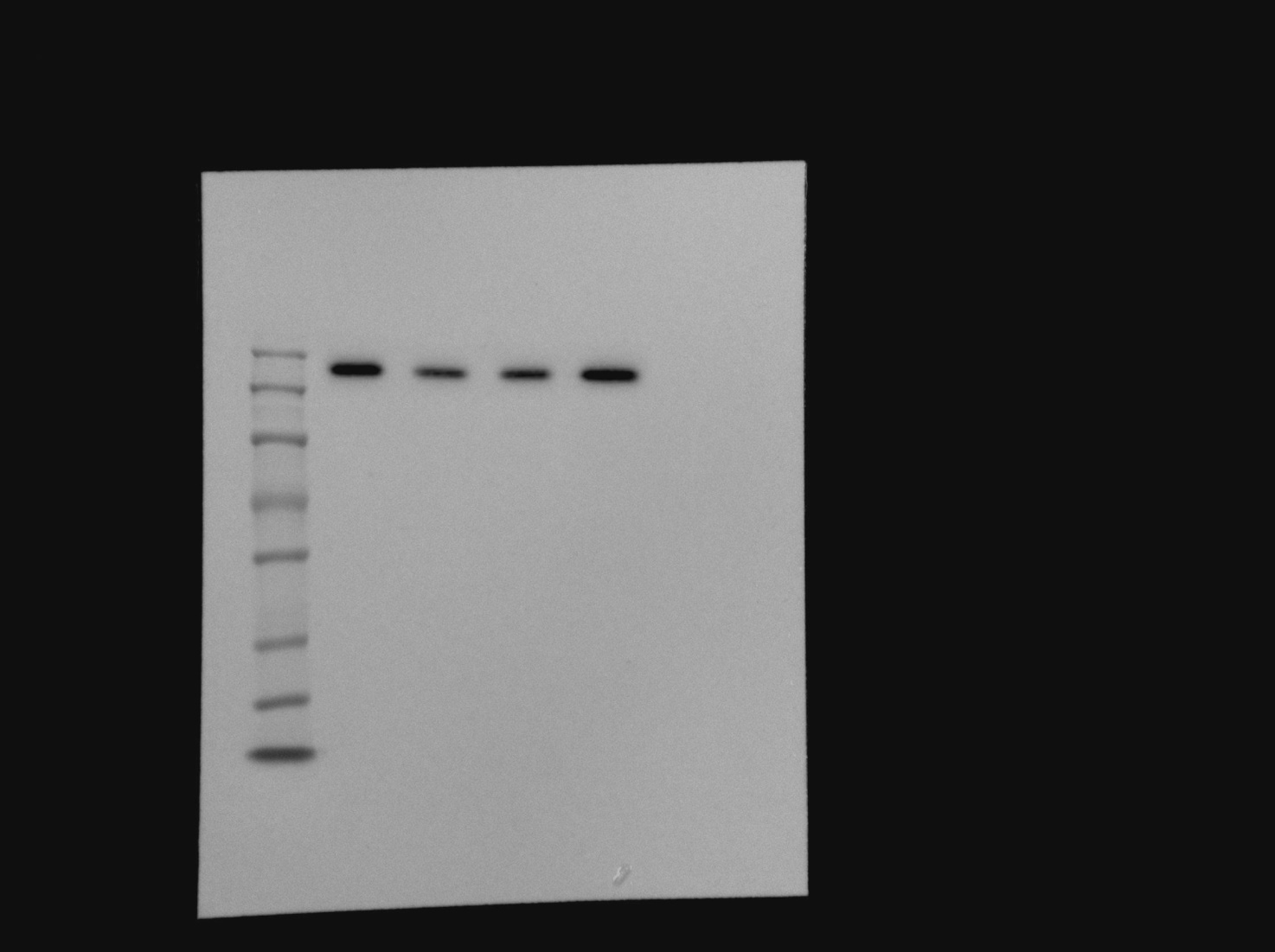


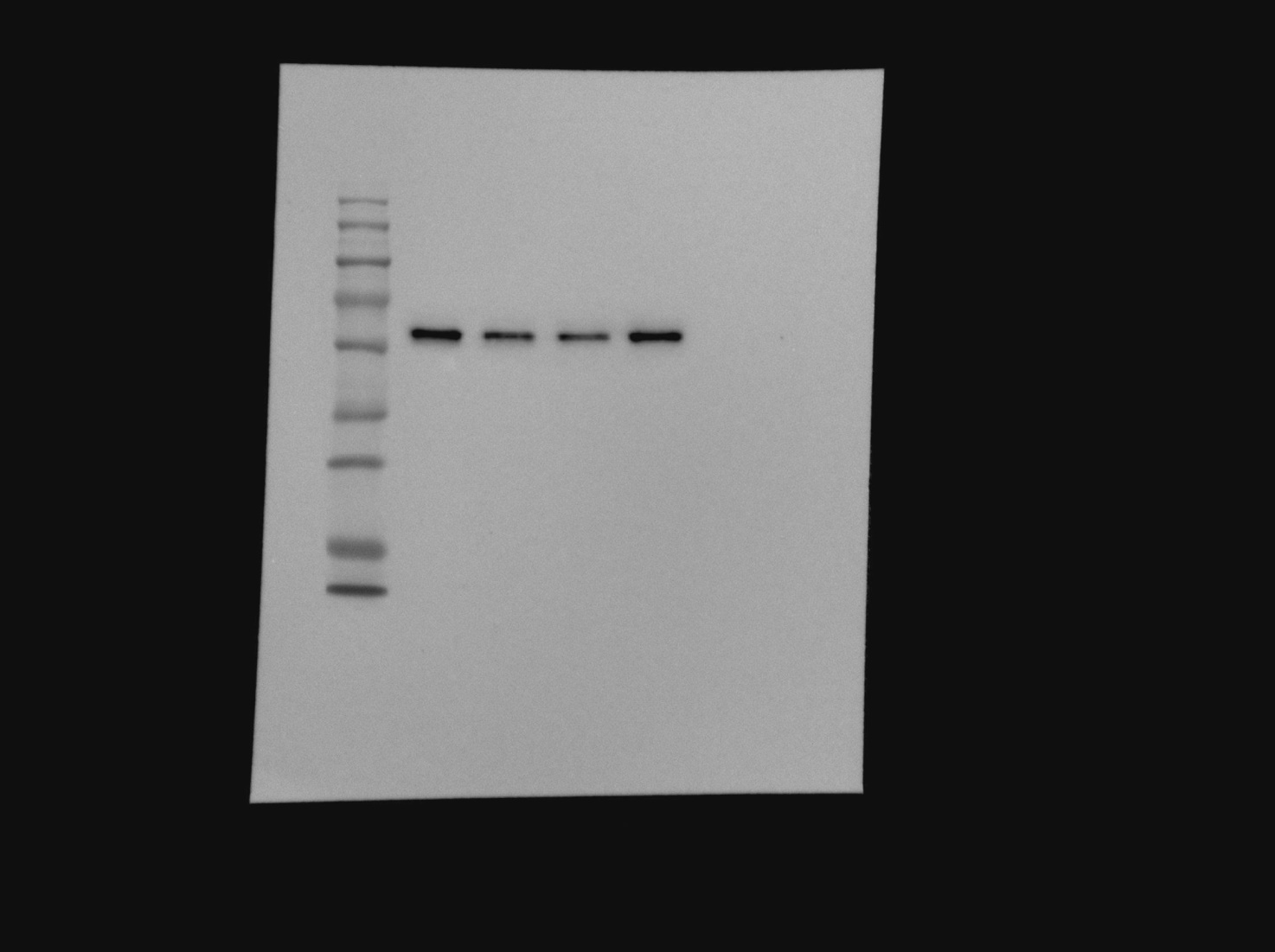


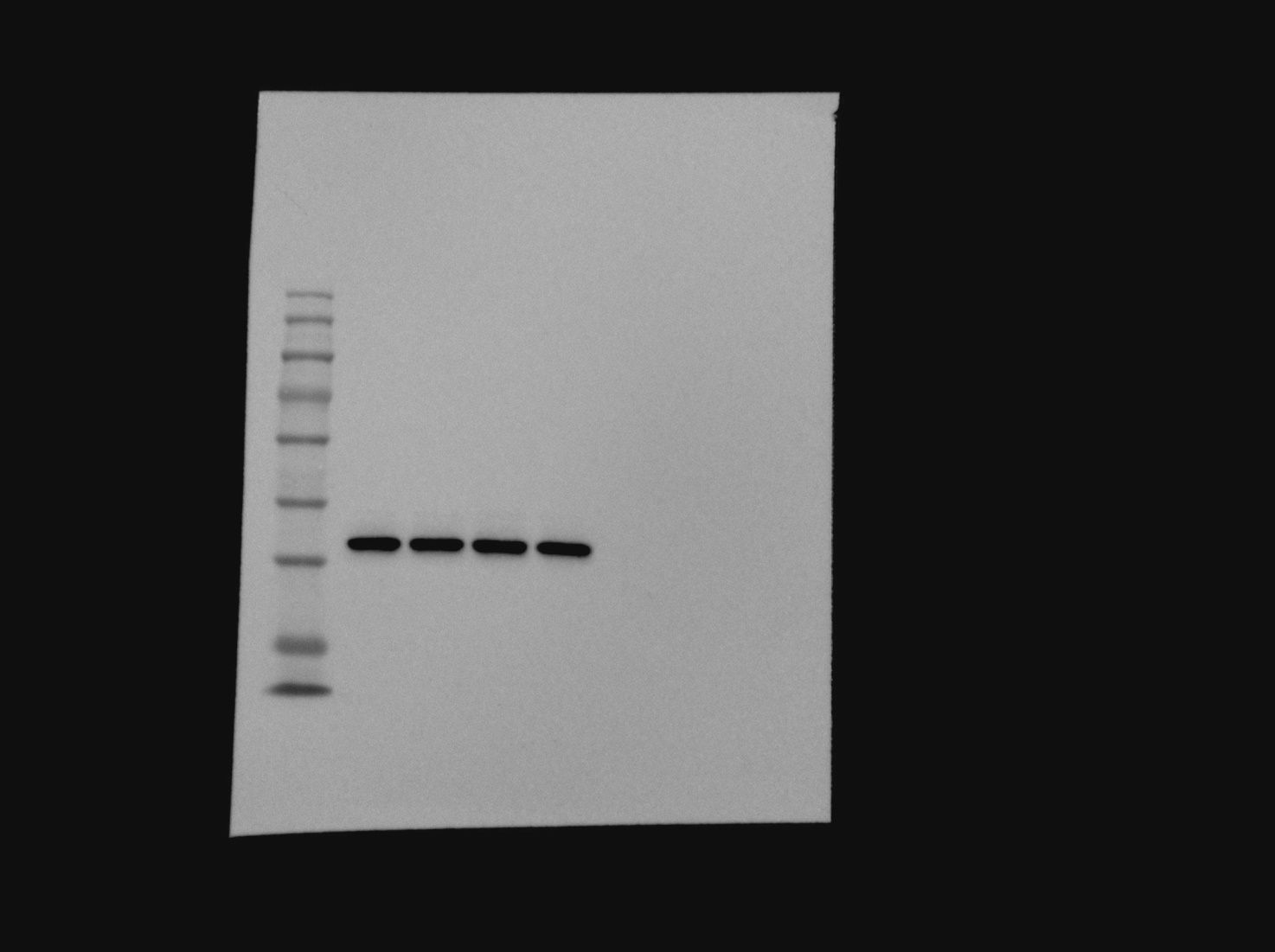


Figure 3D


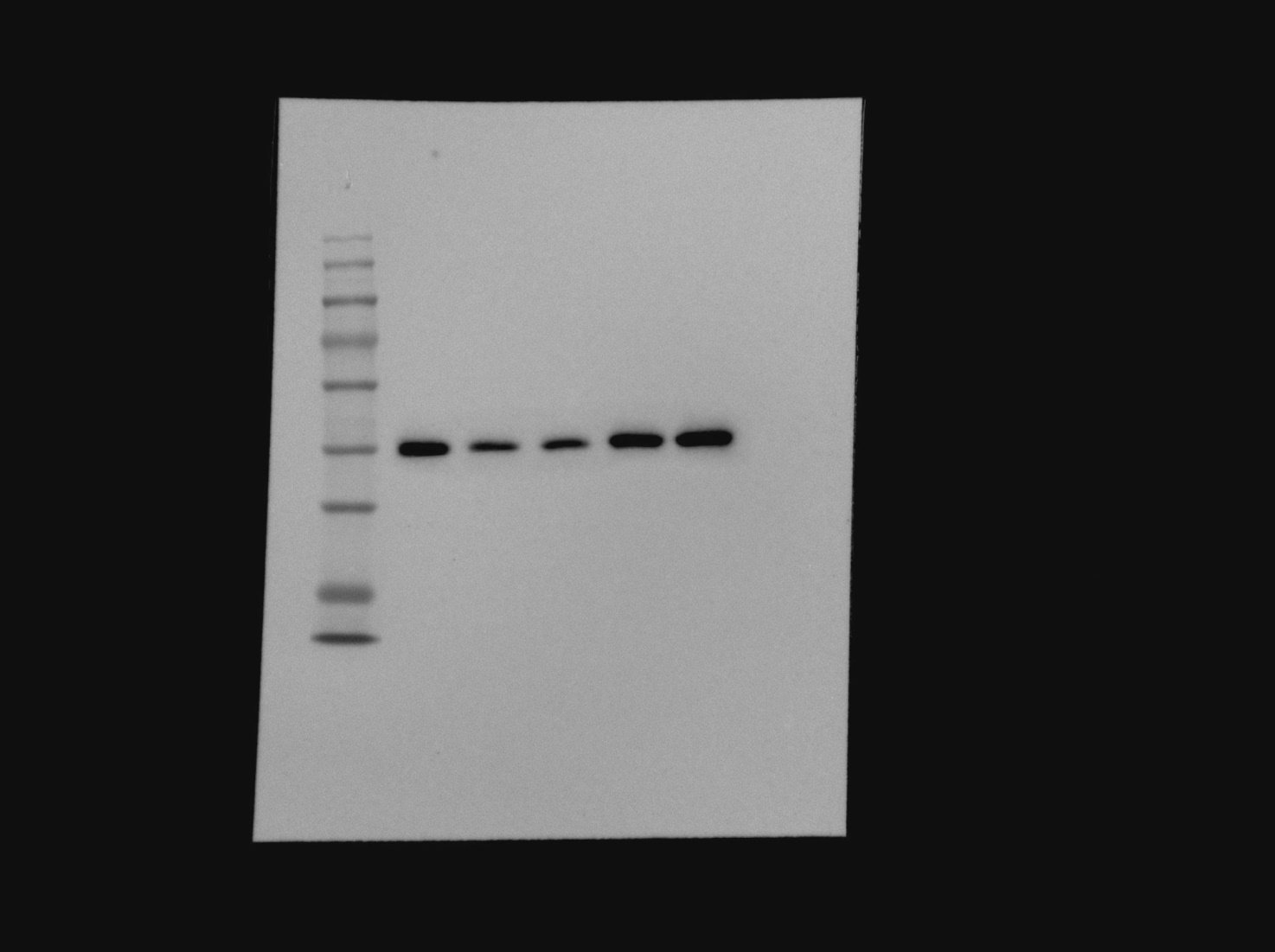


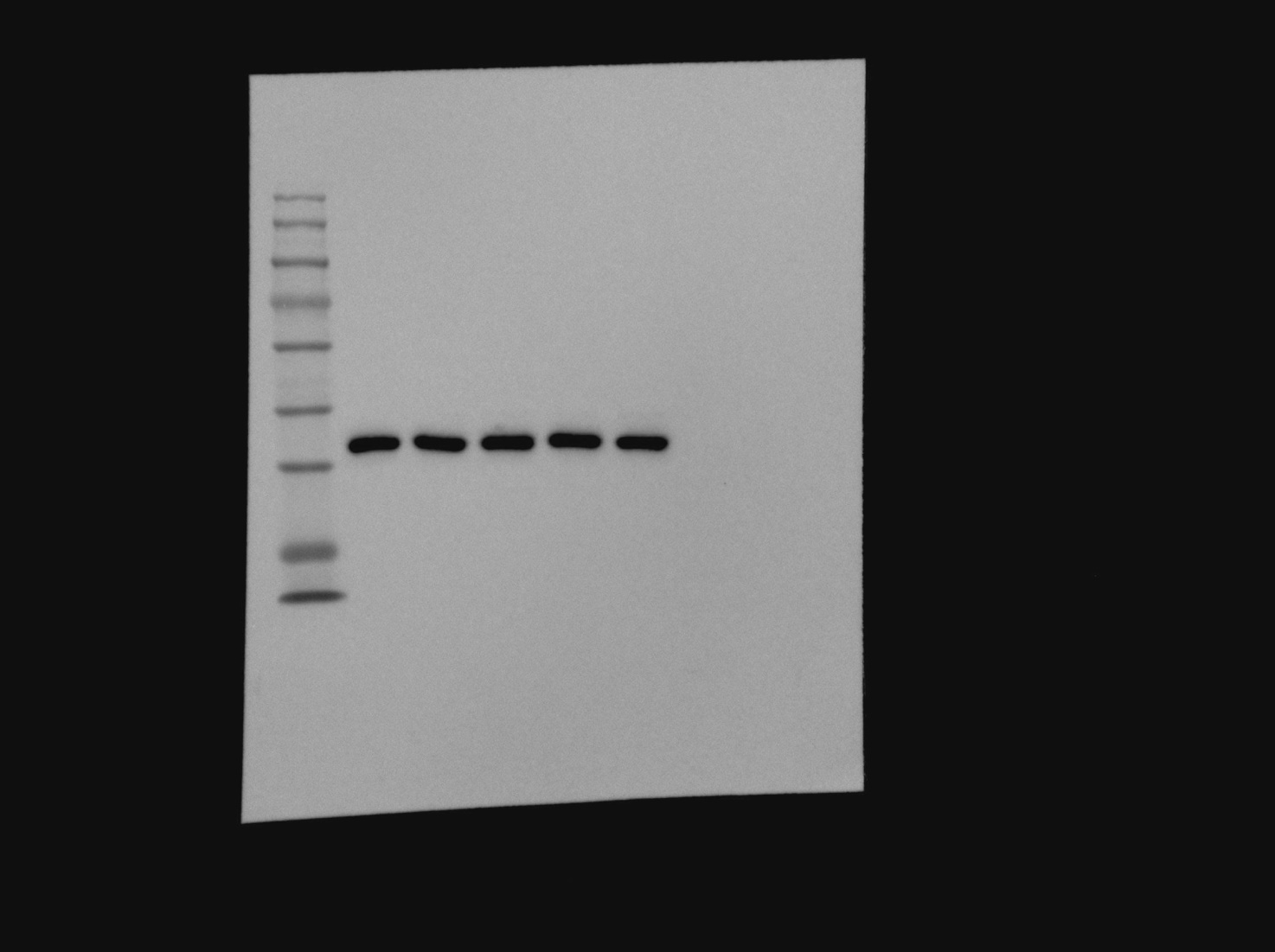


Figure3E


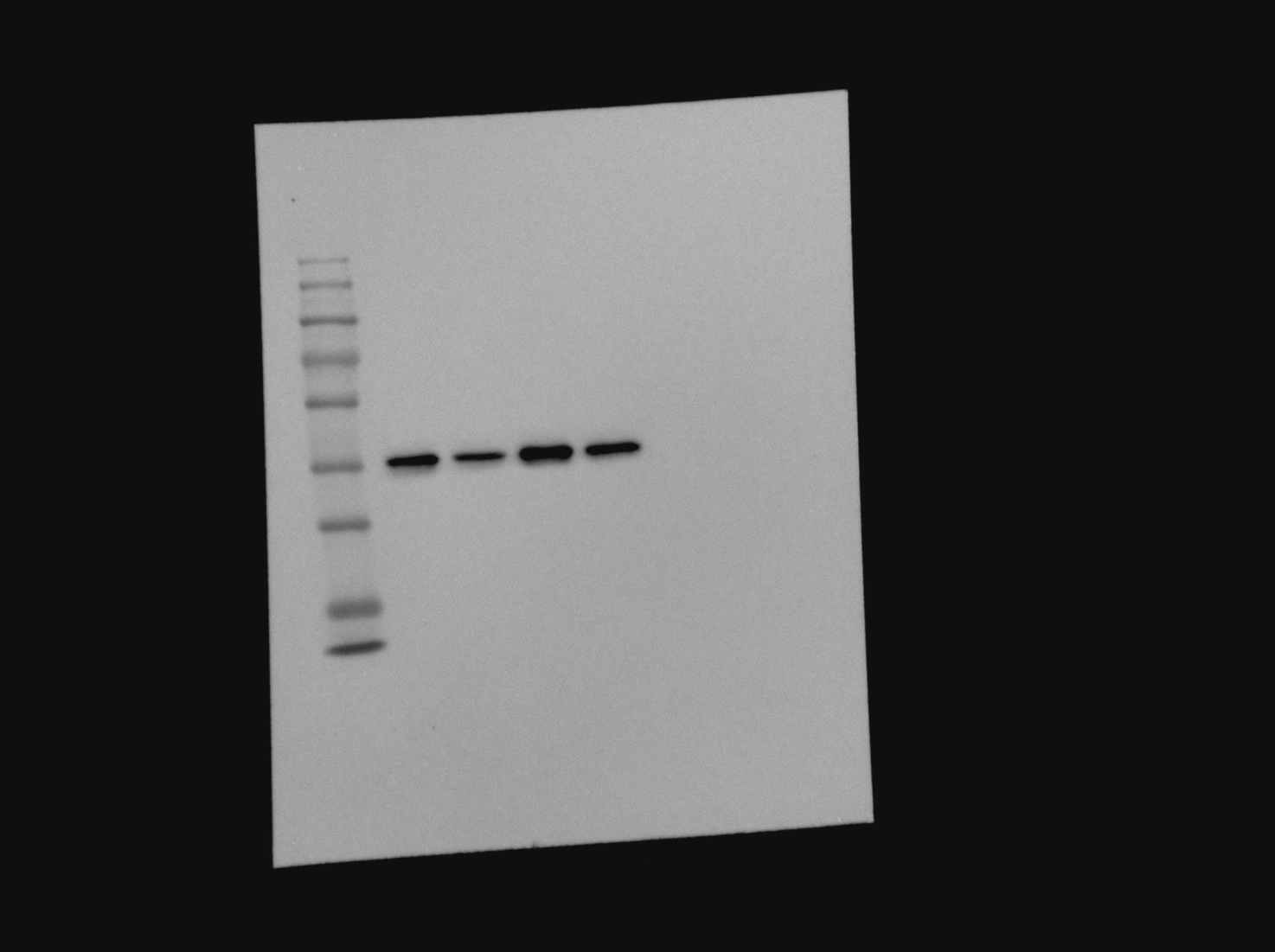


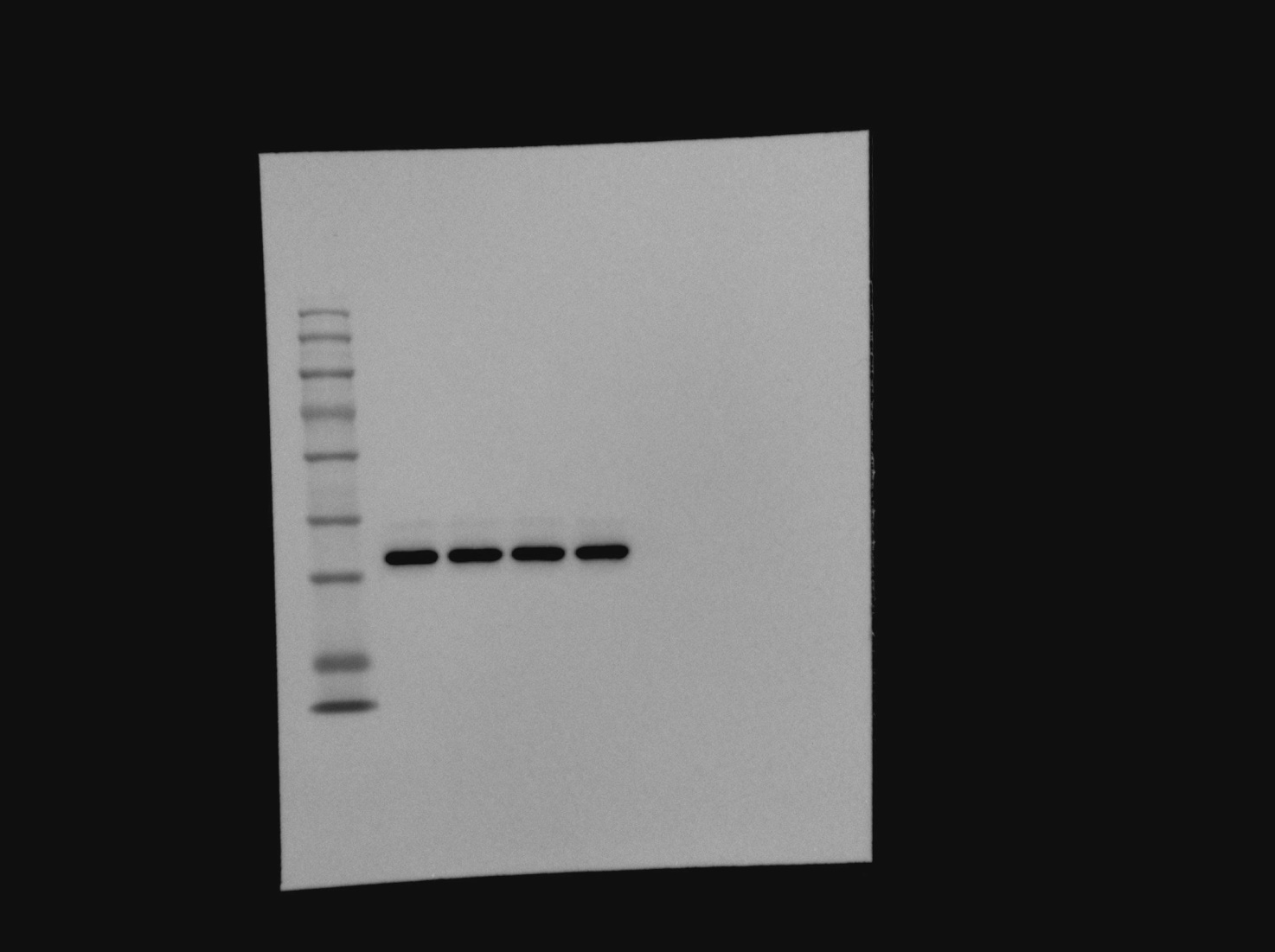


Figure4A


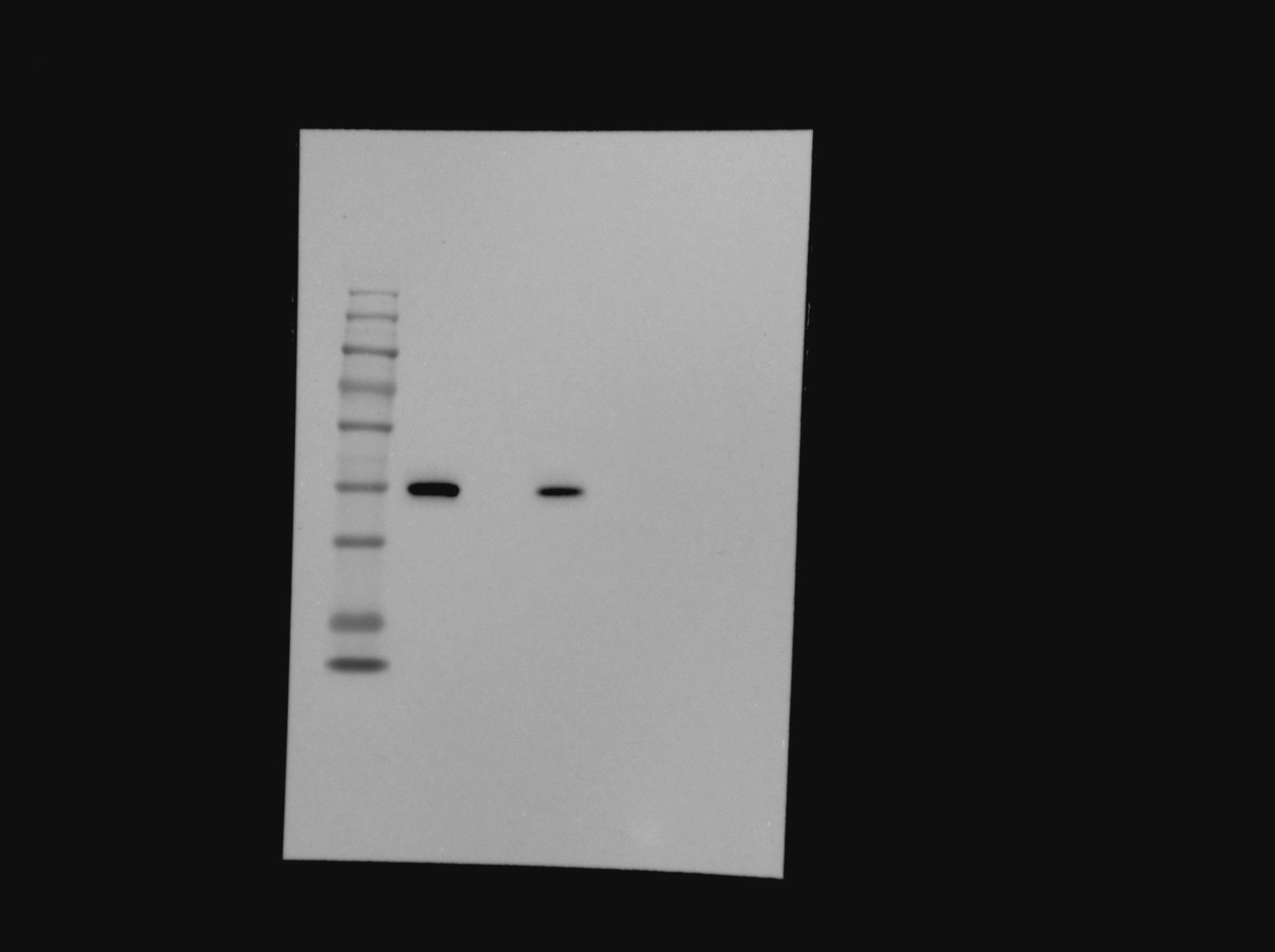


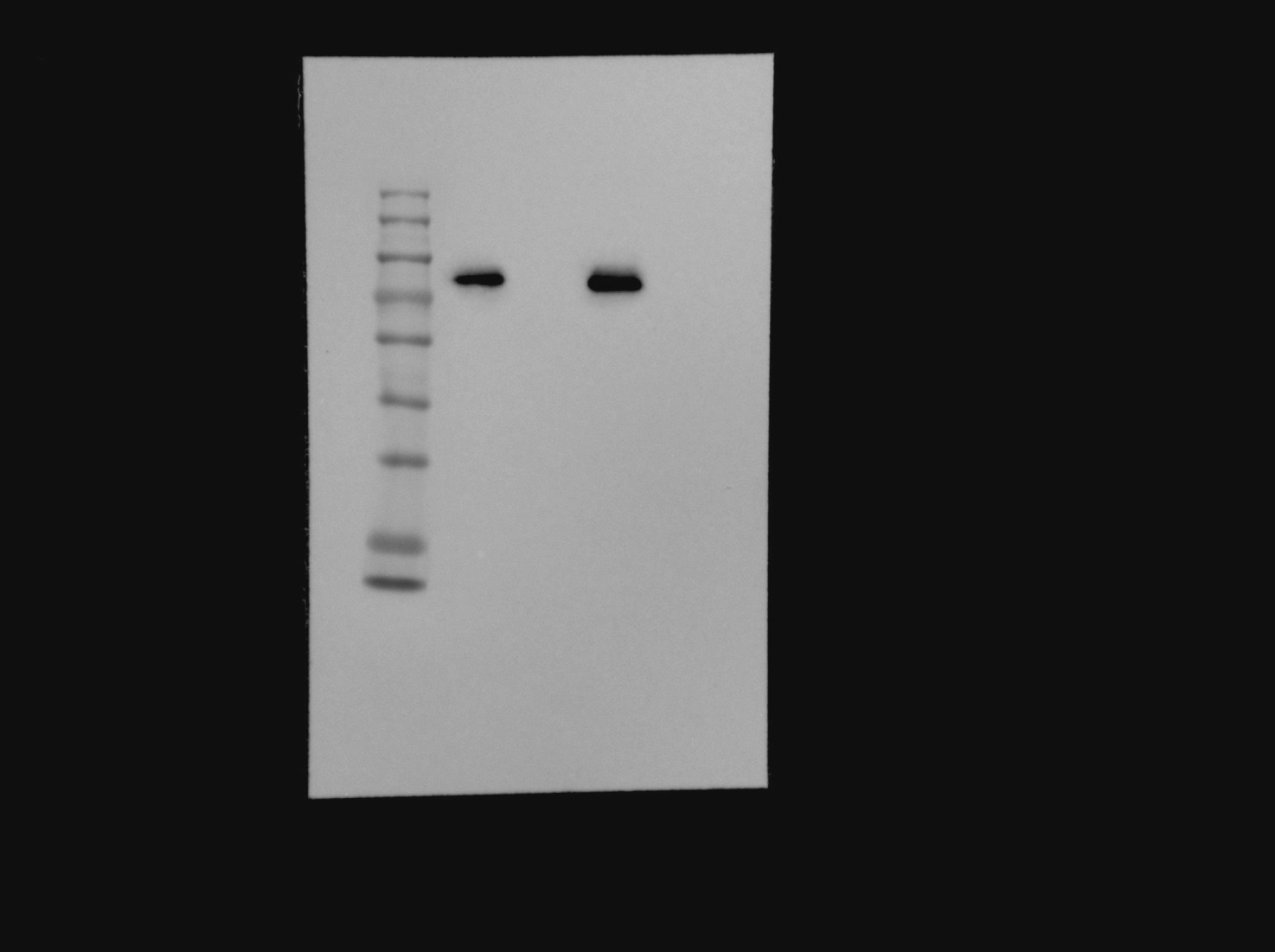


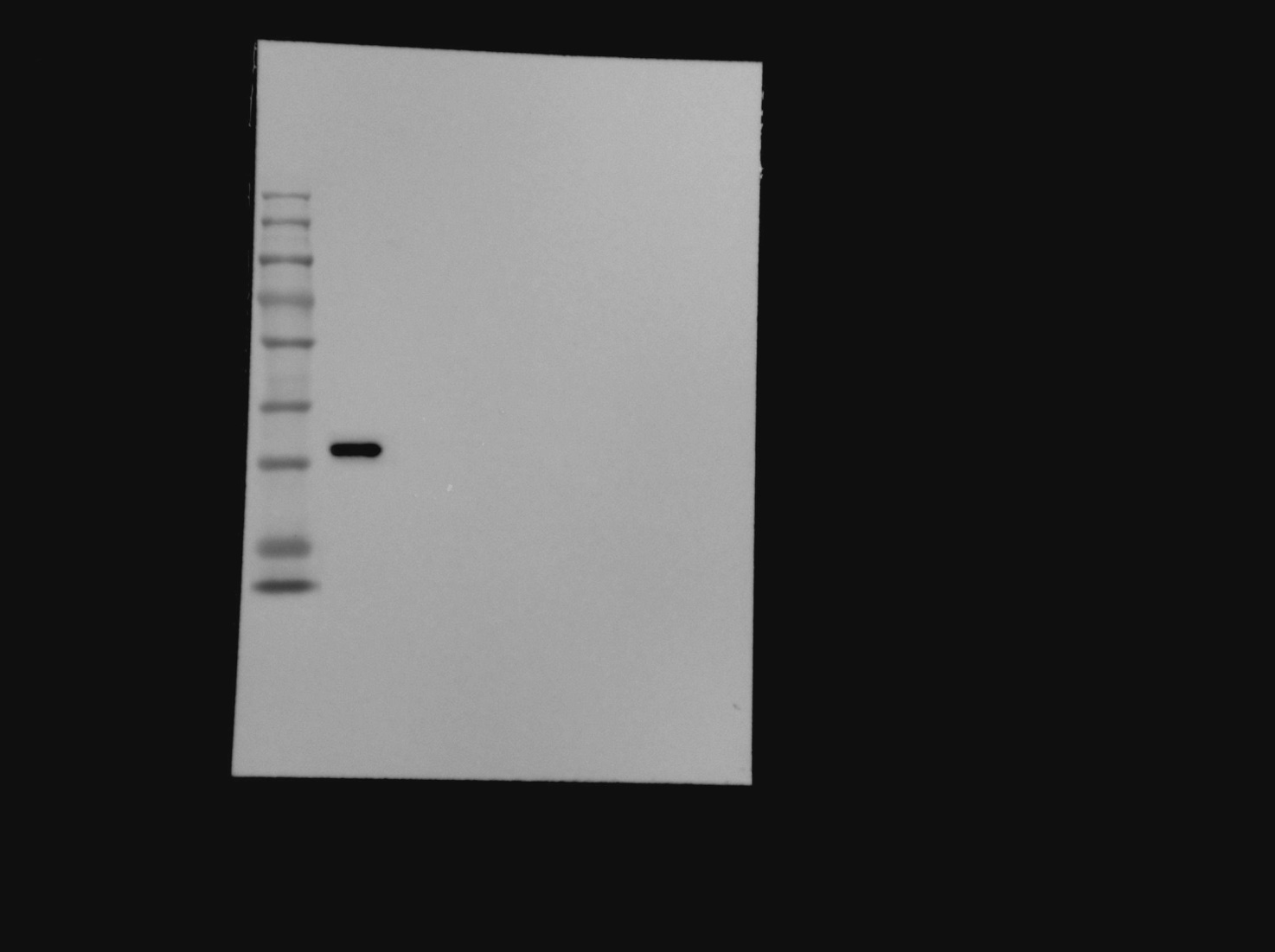


Figure4C


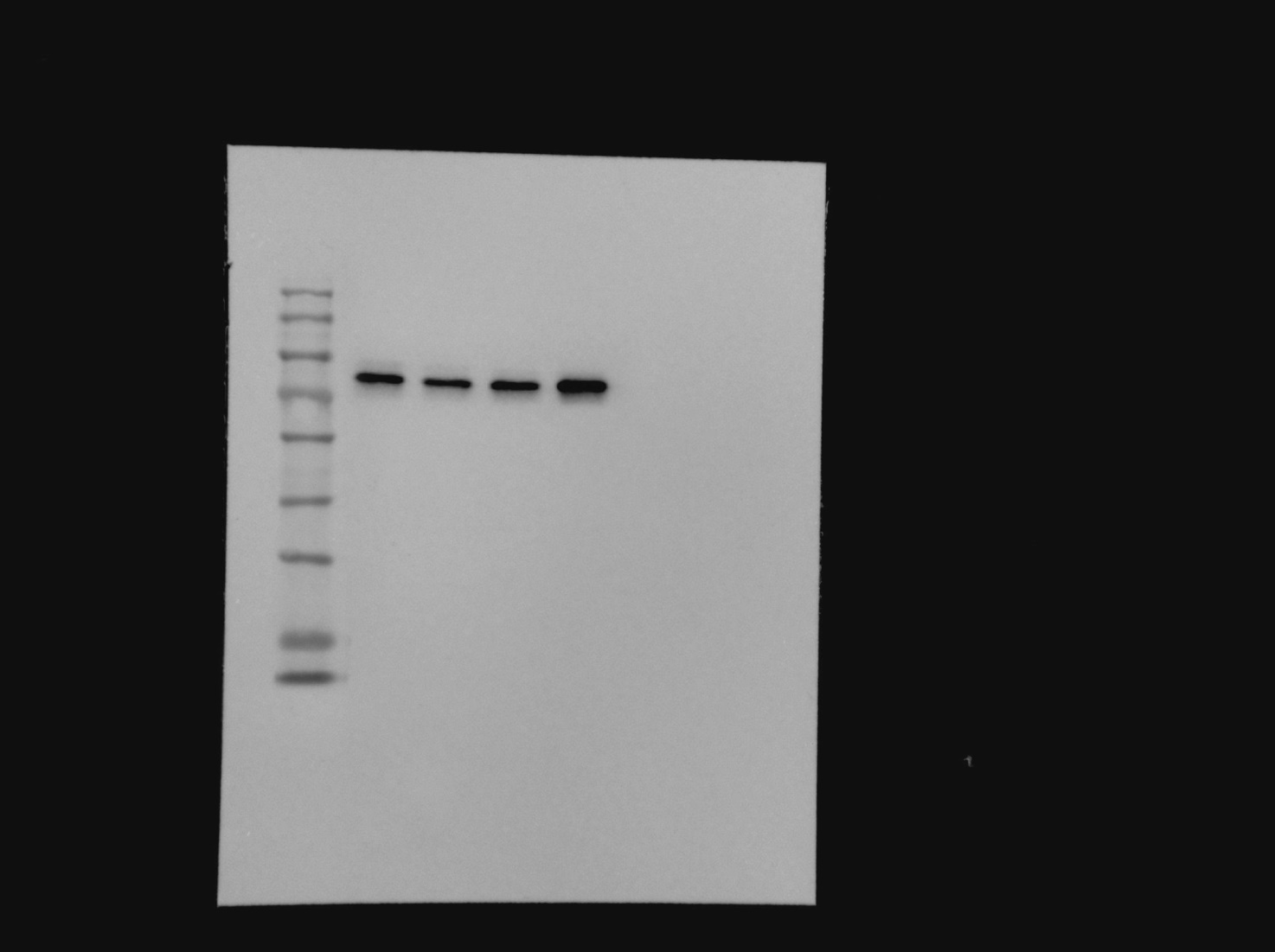


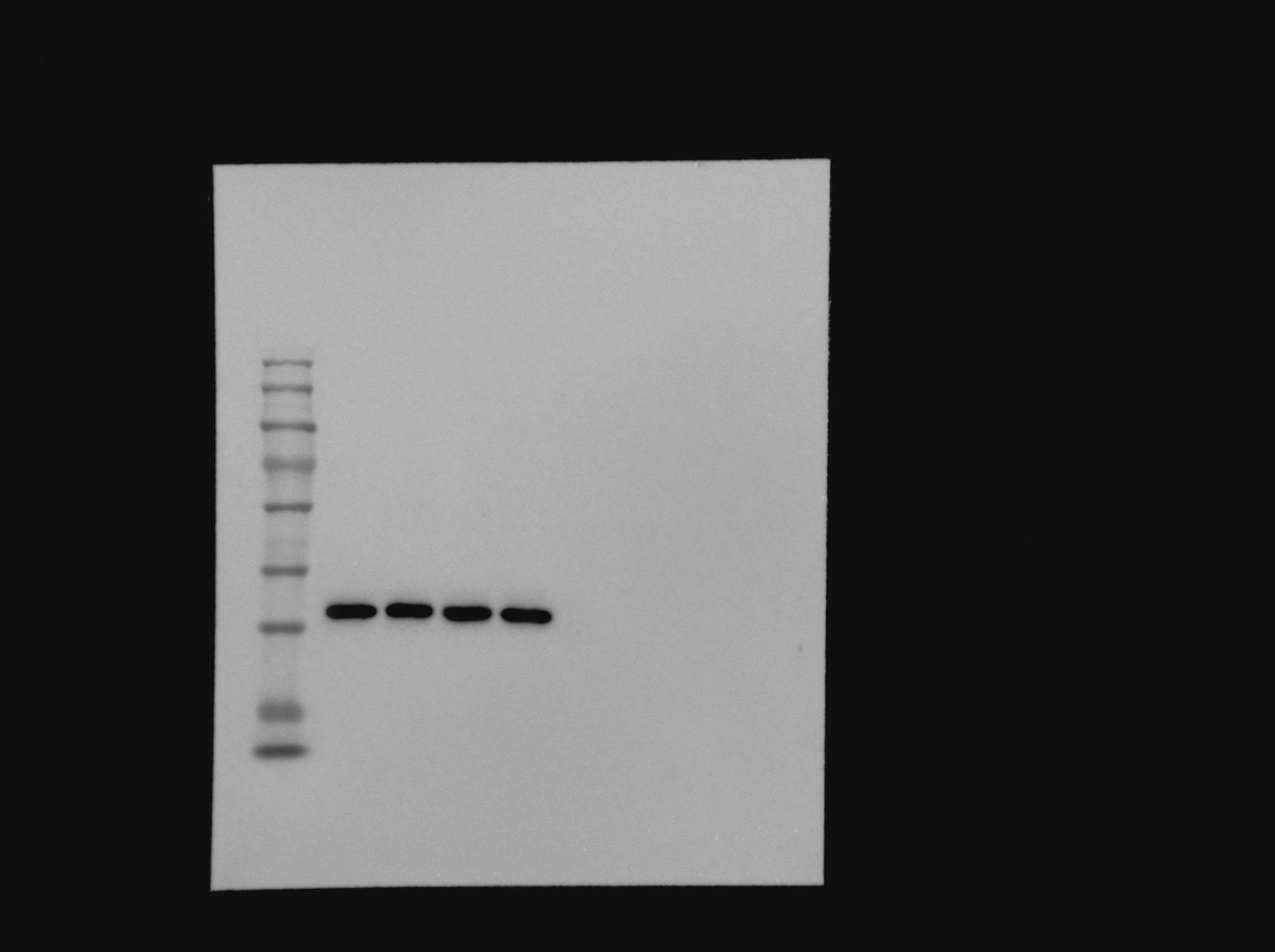


Figure4D


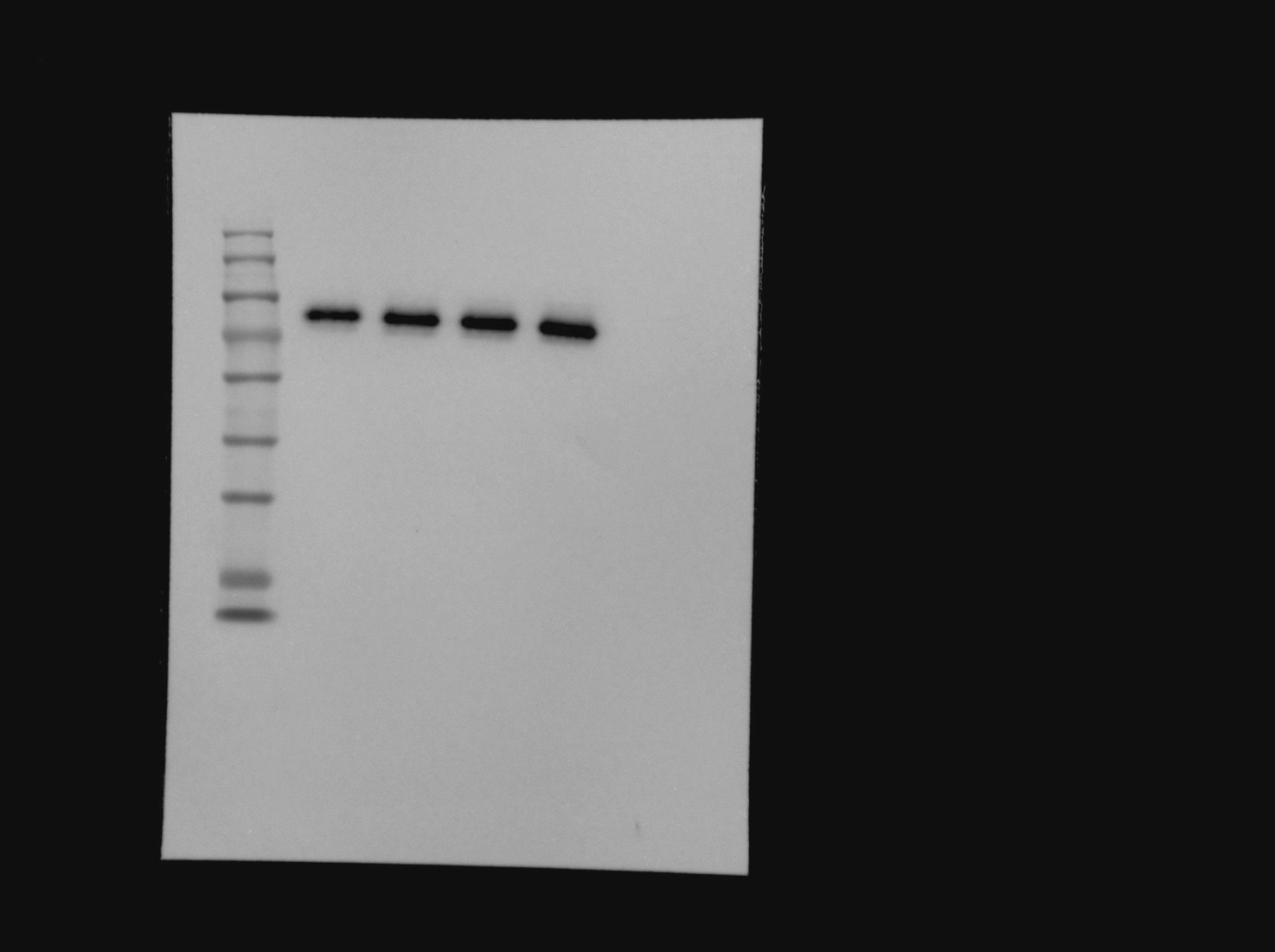


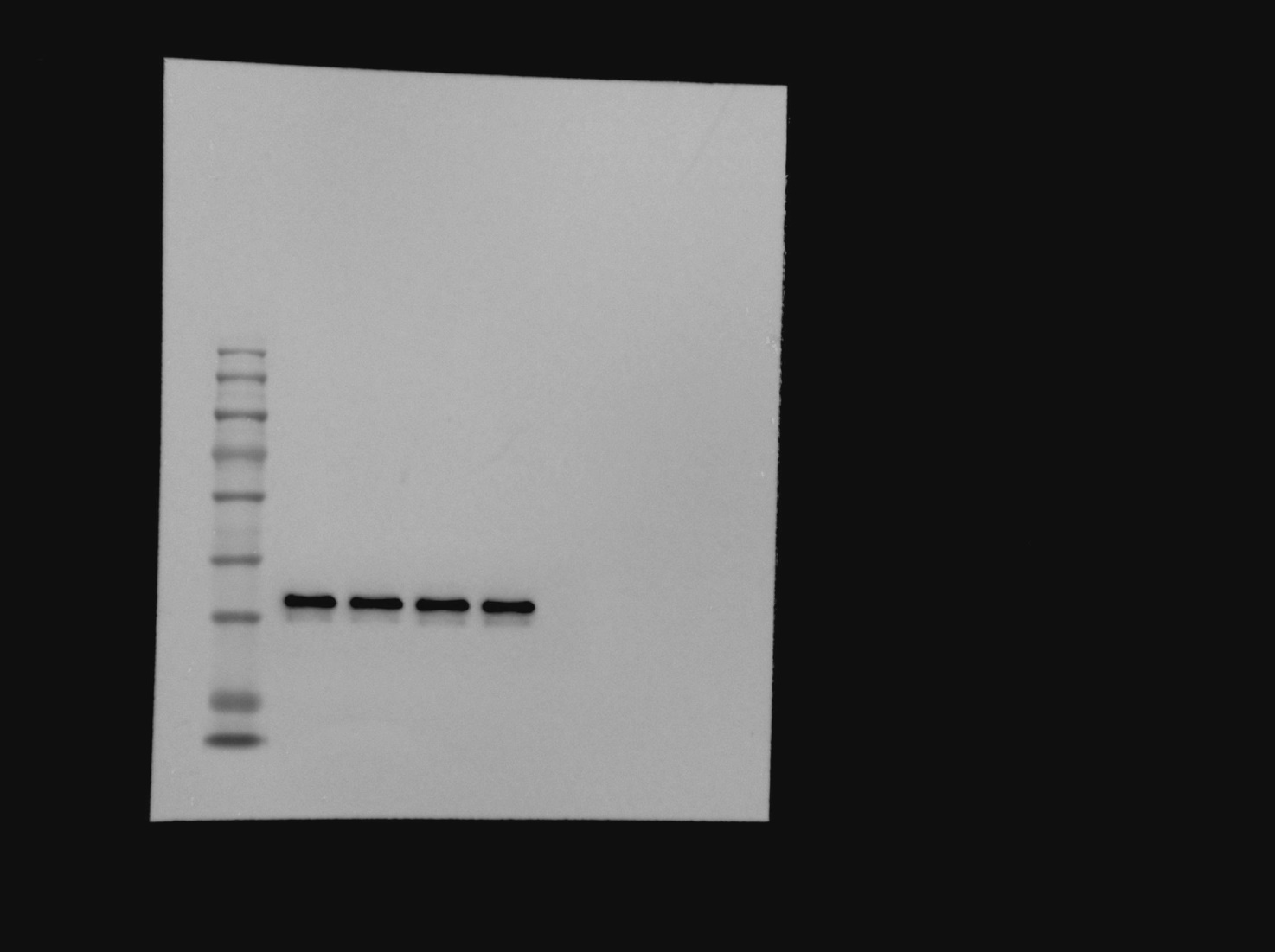


Figure5C


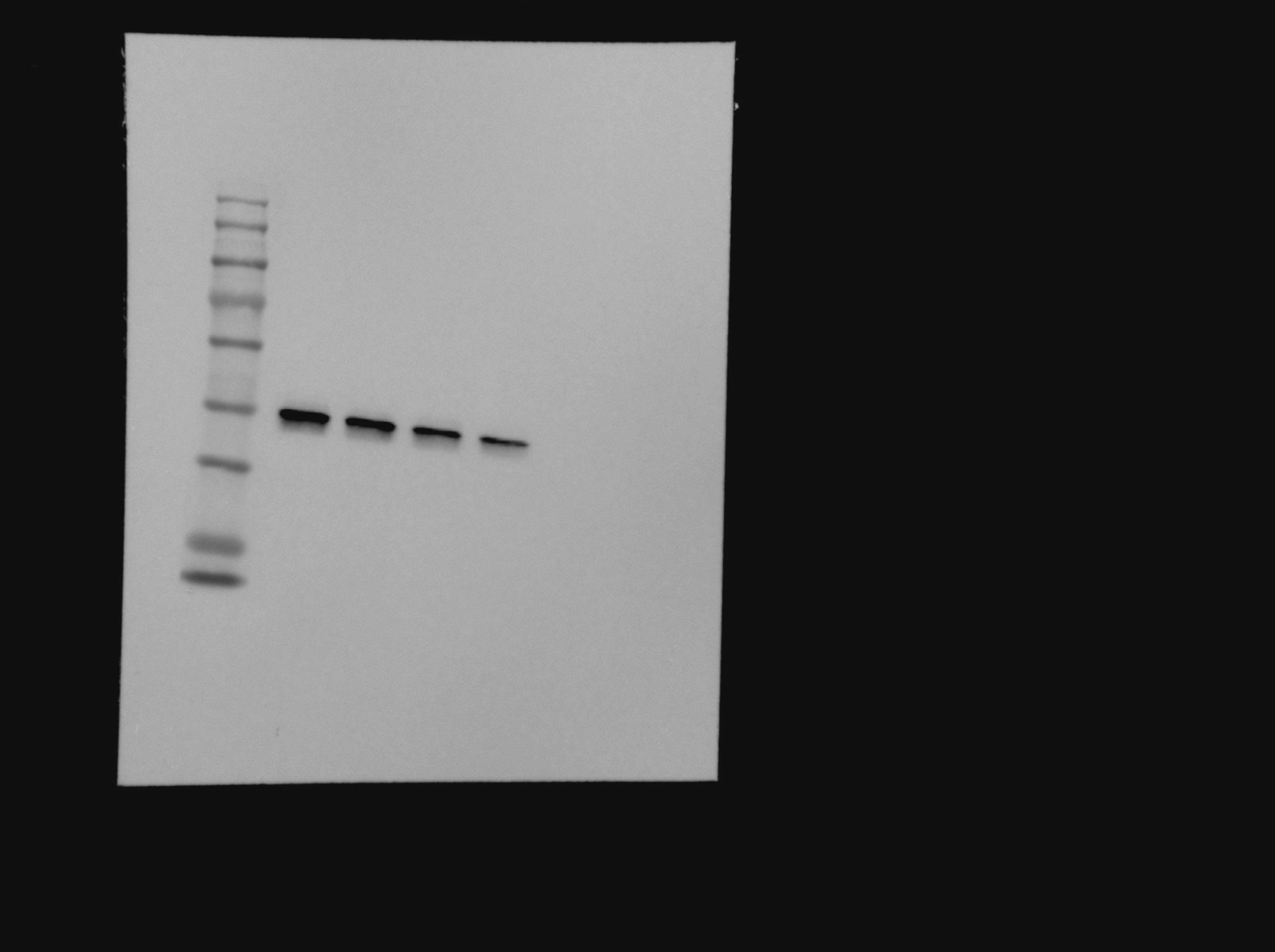


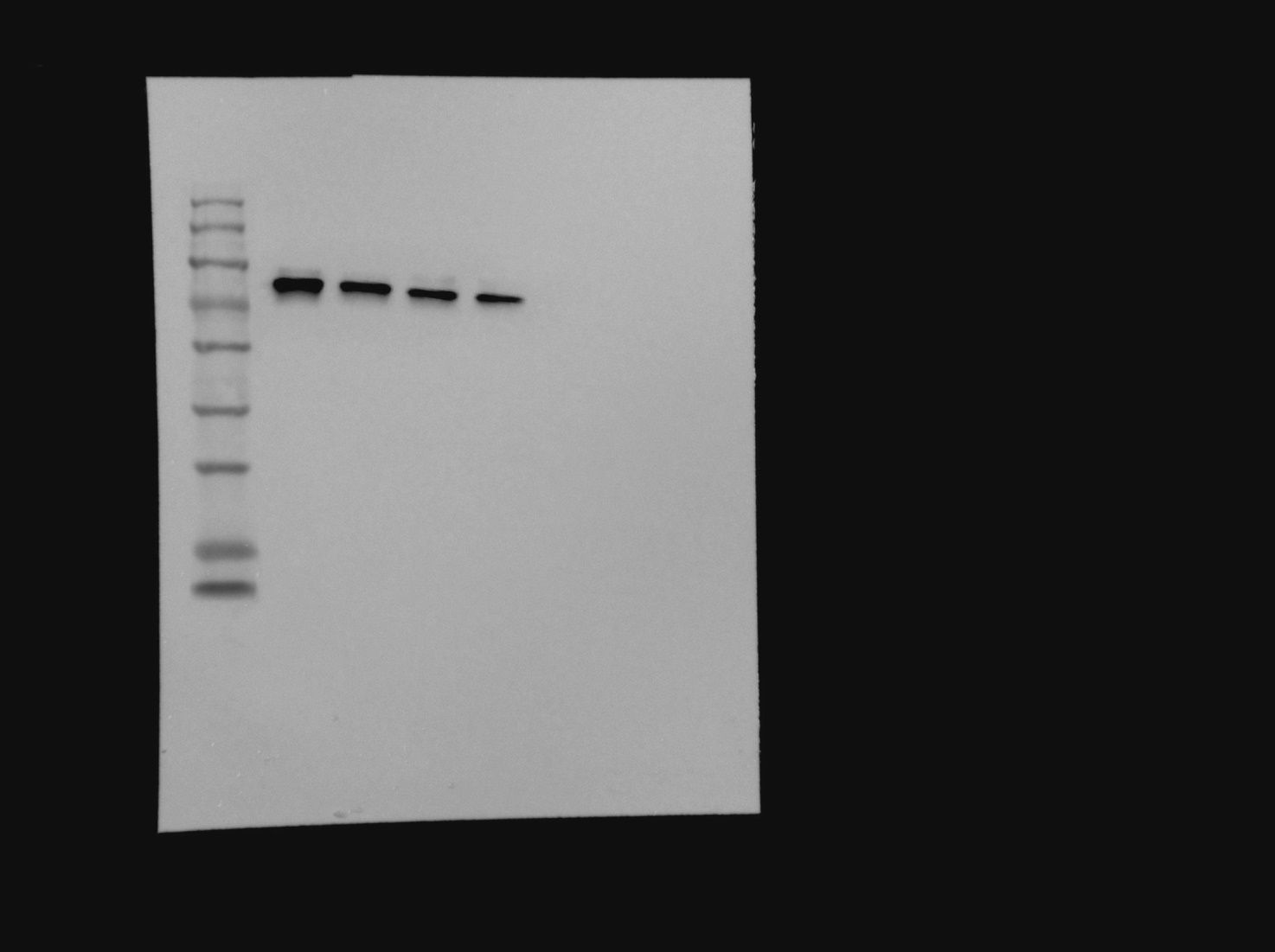


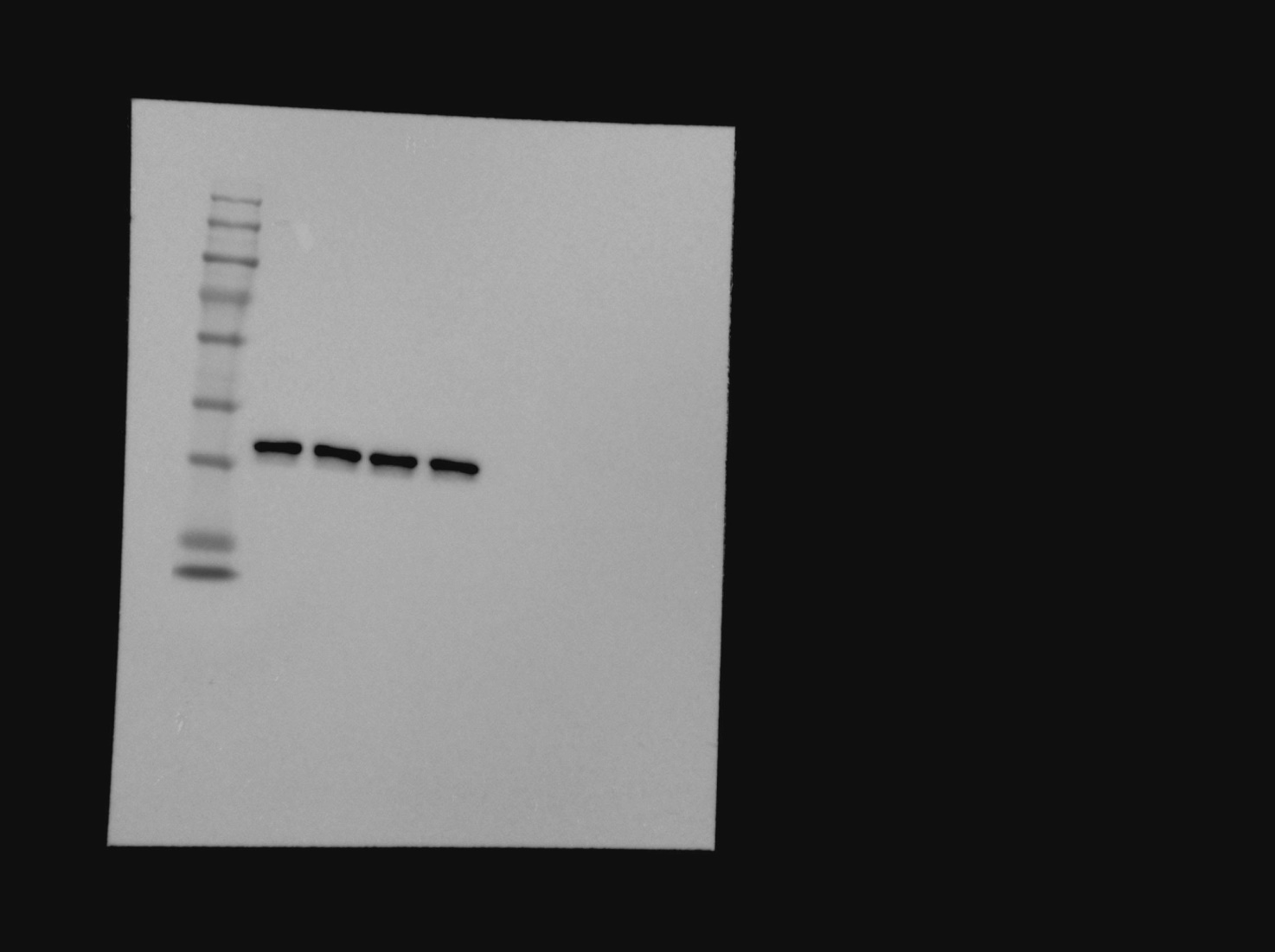


Figure5J


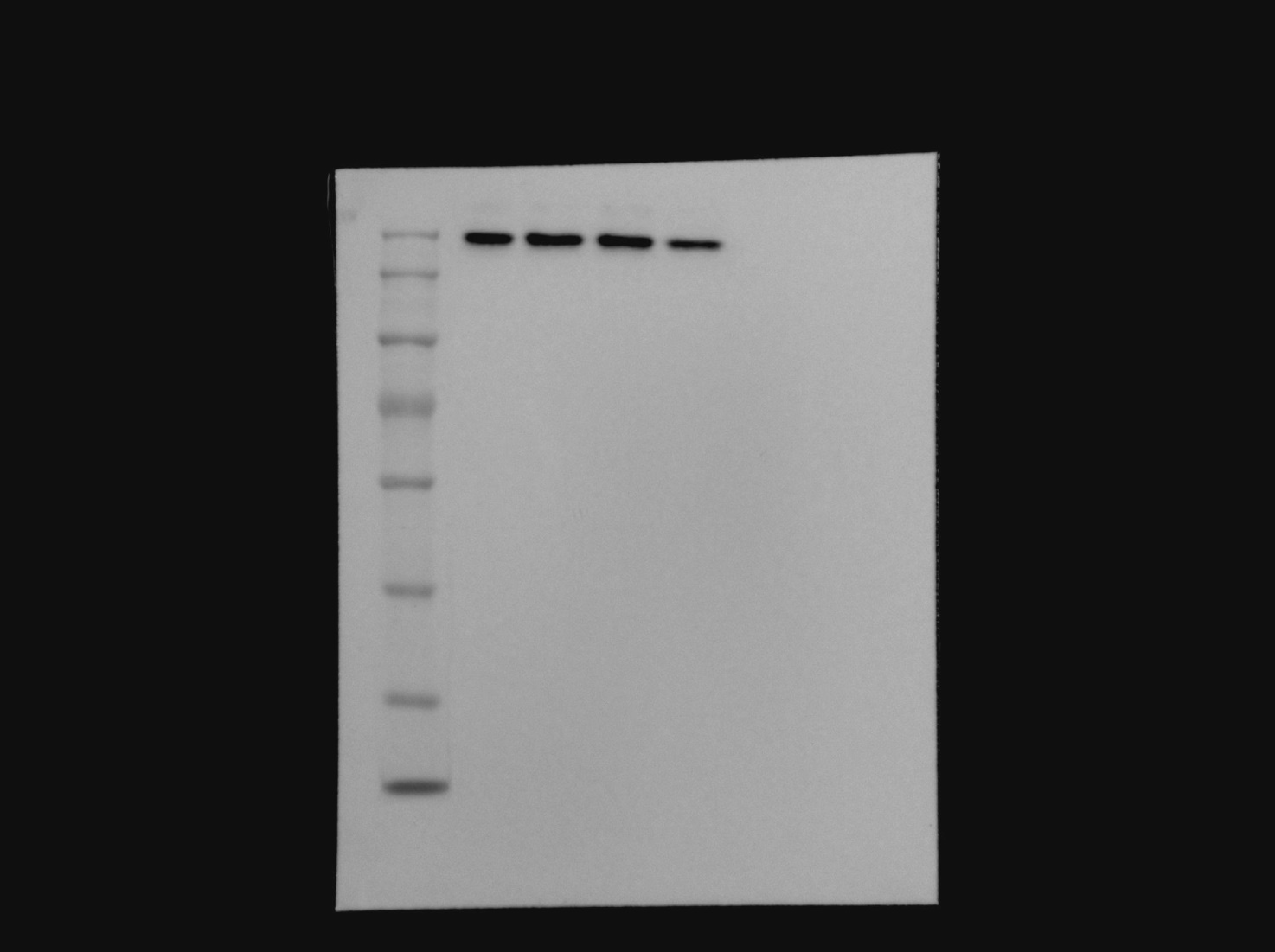


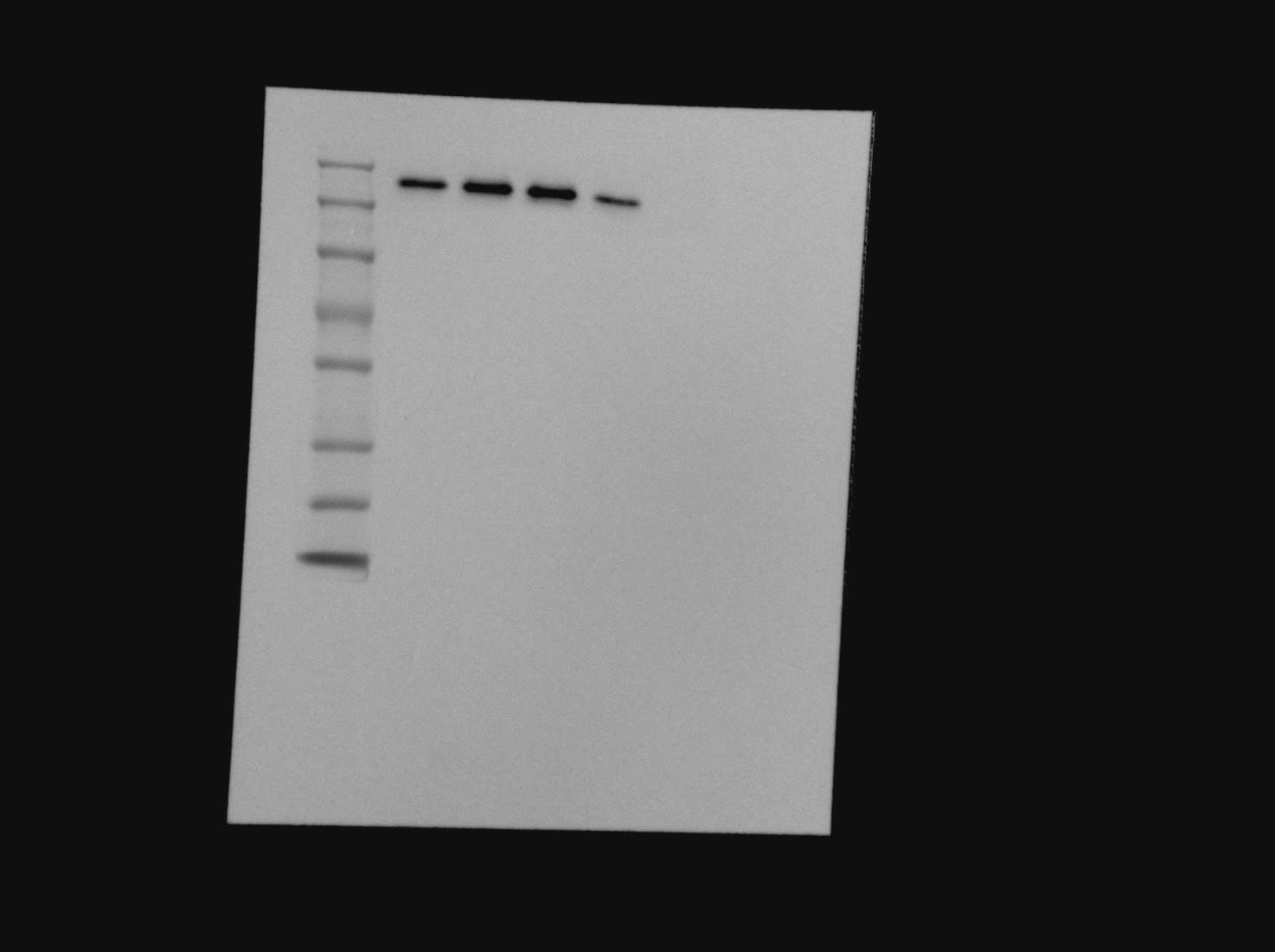


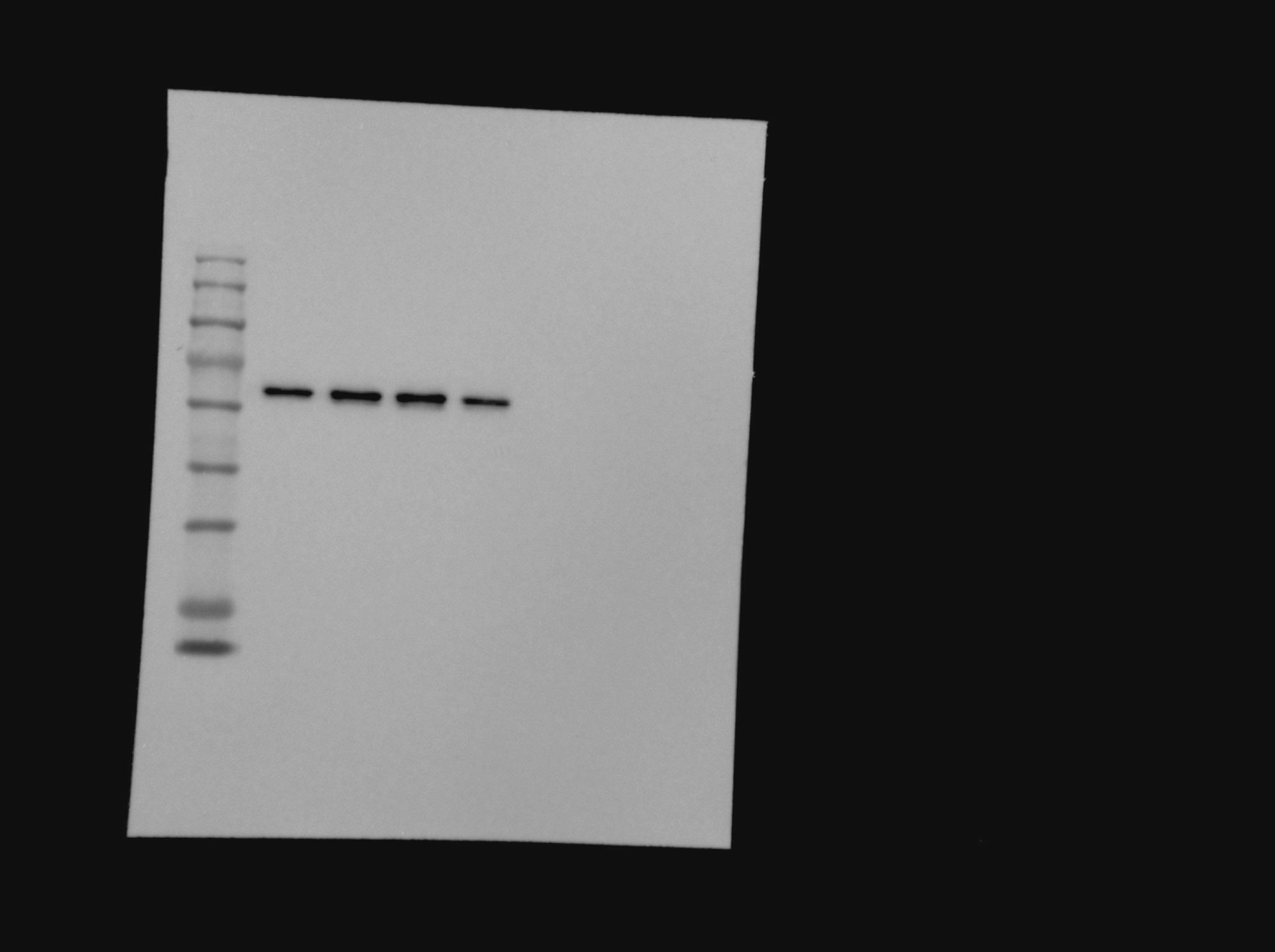


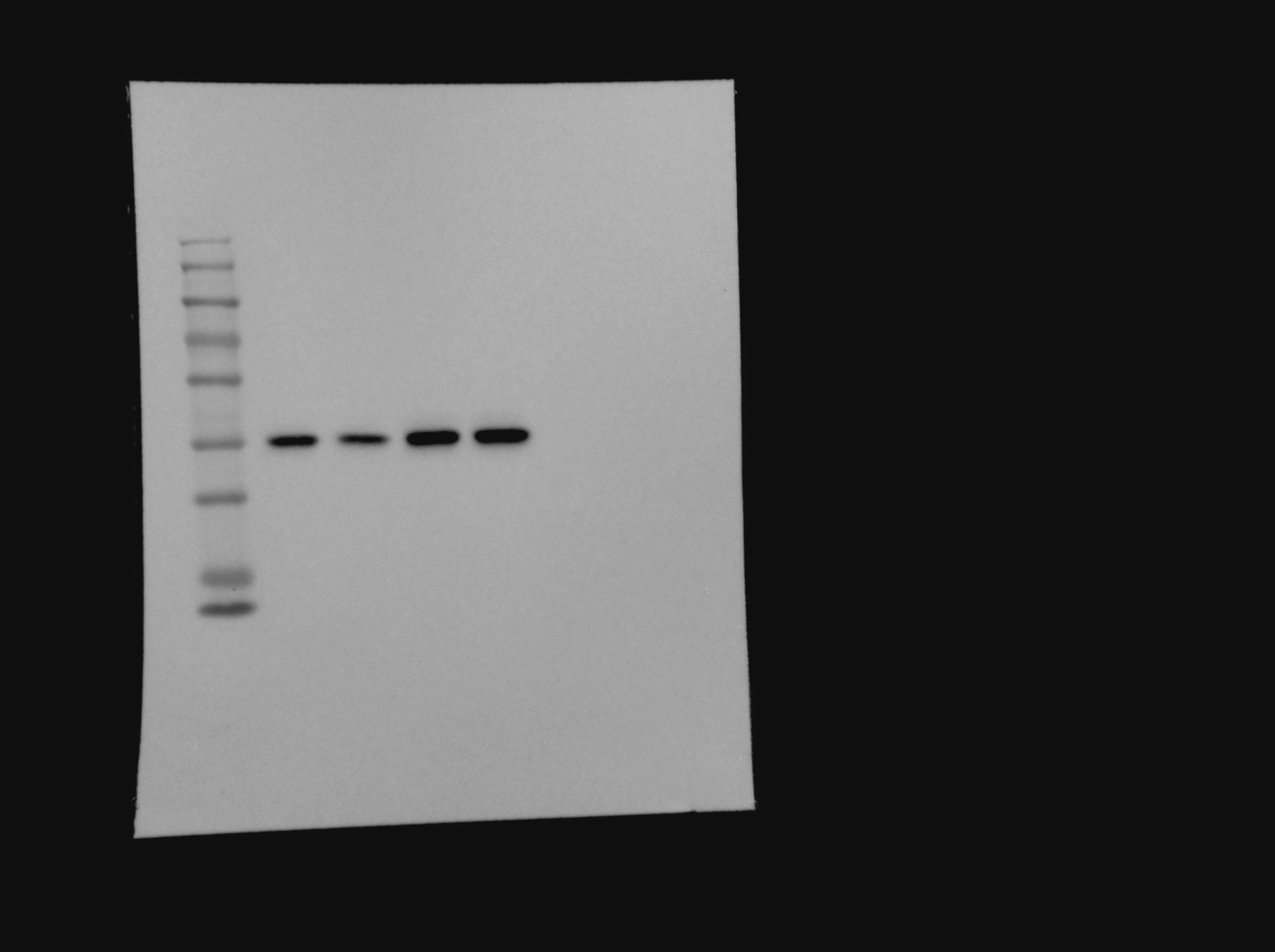


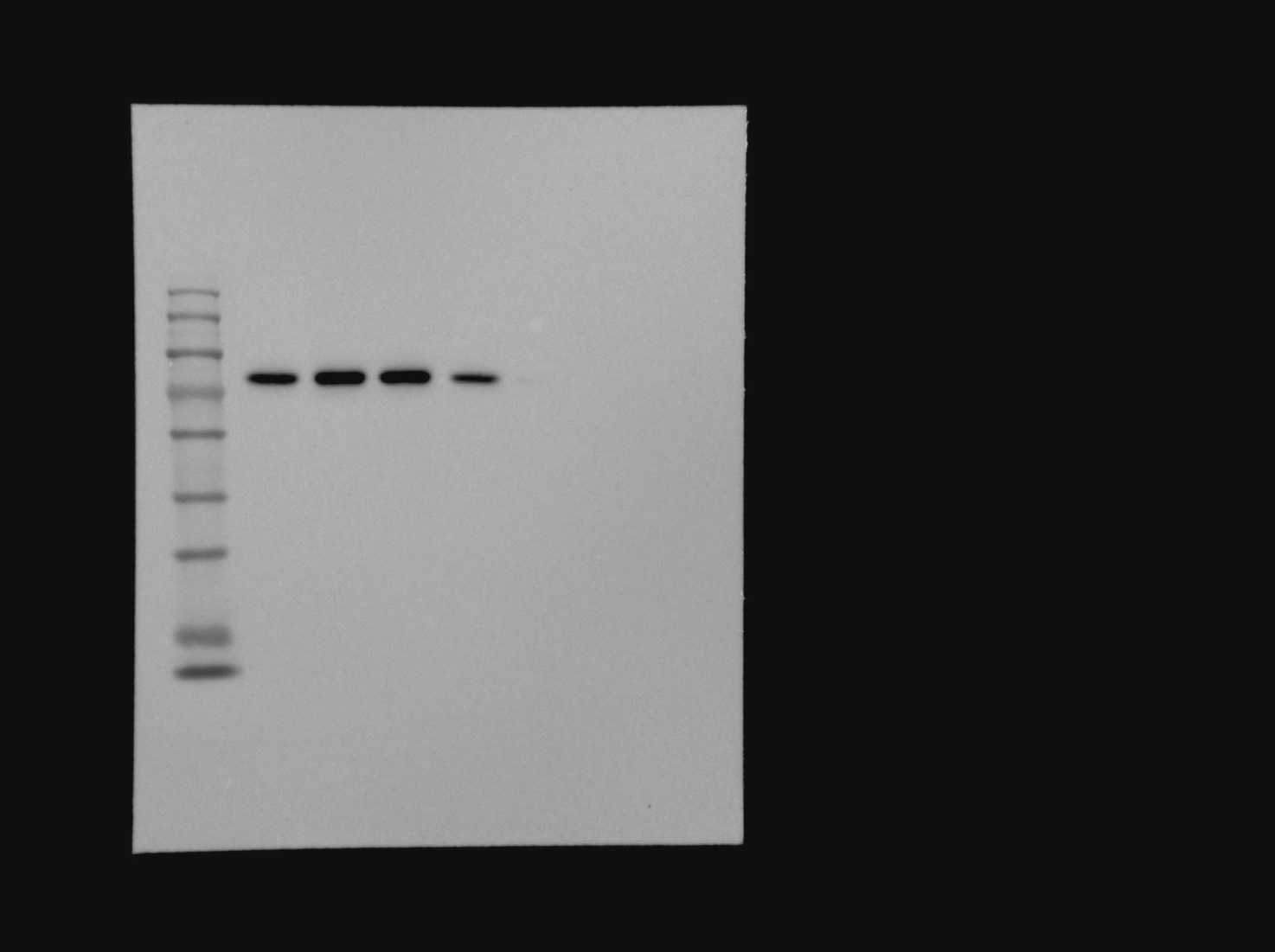


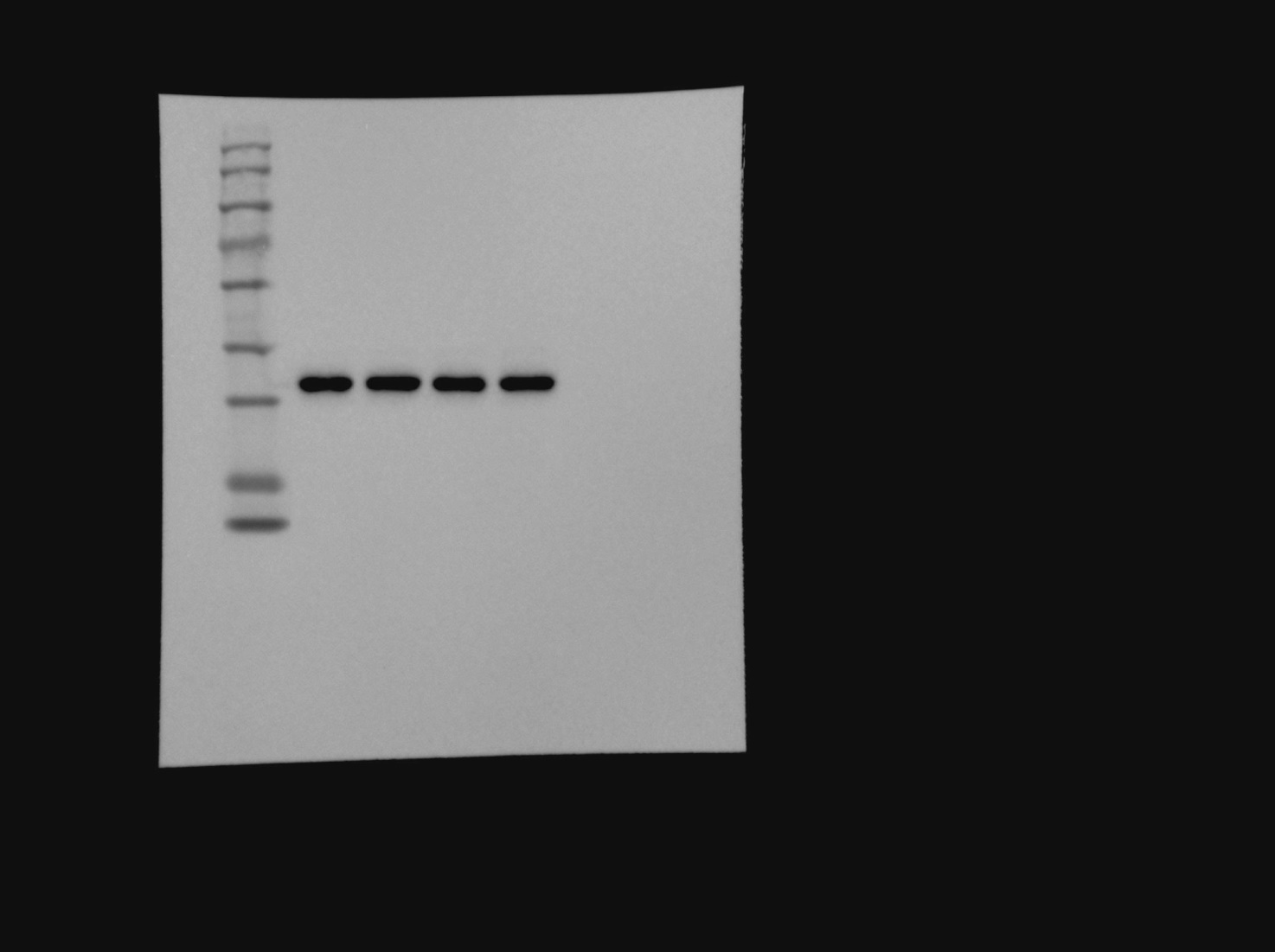


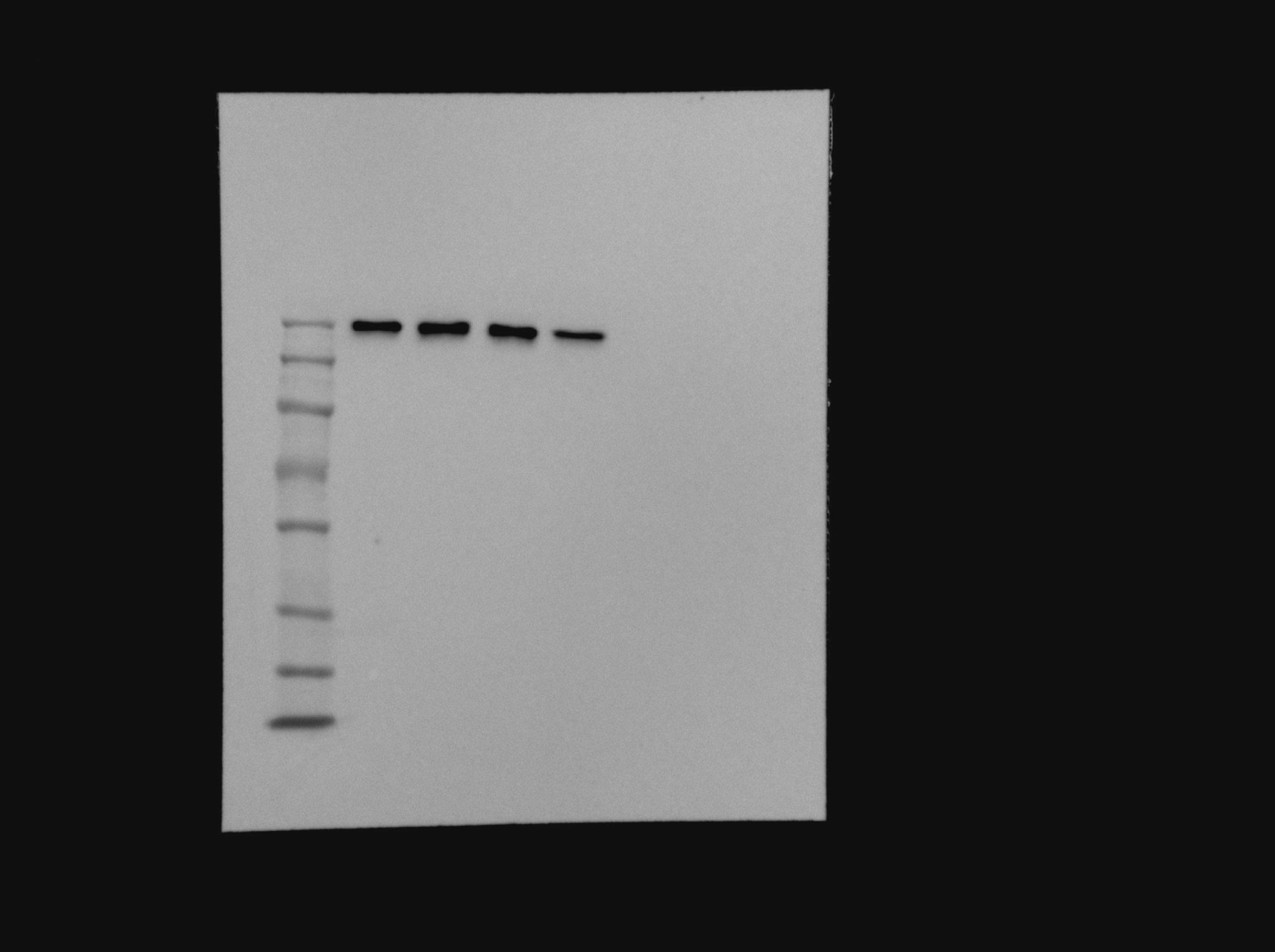


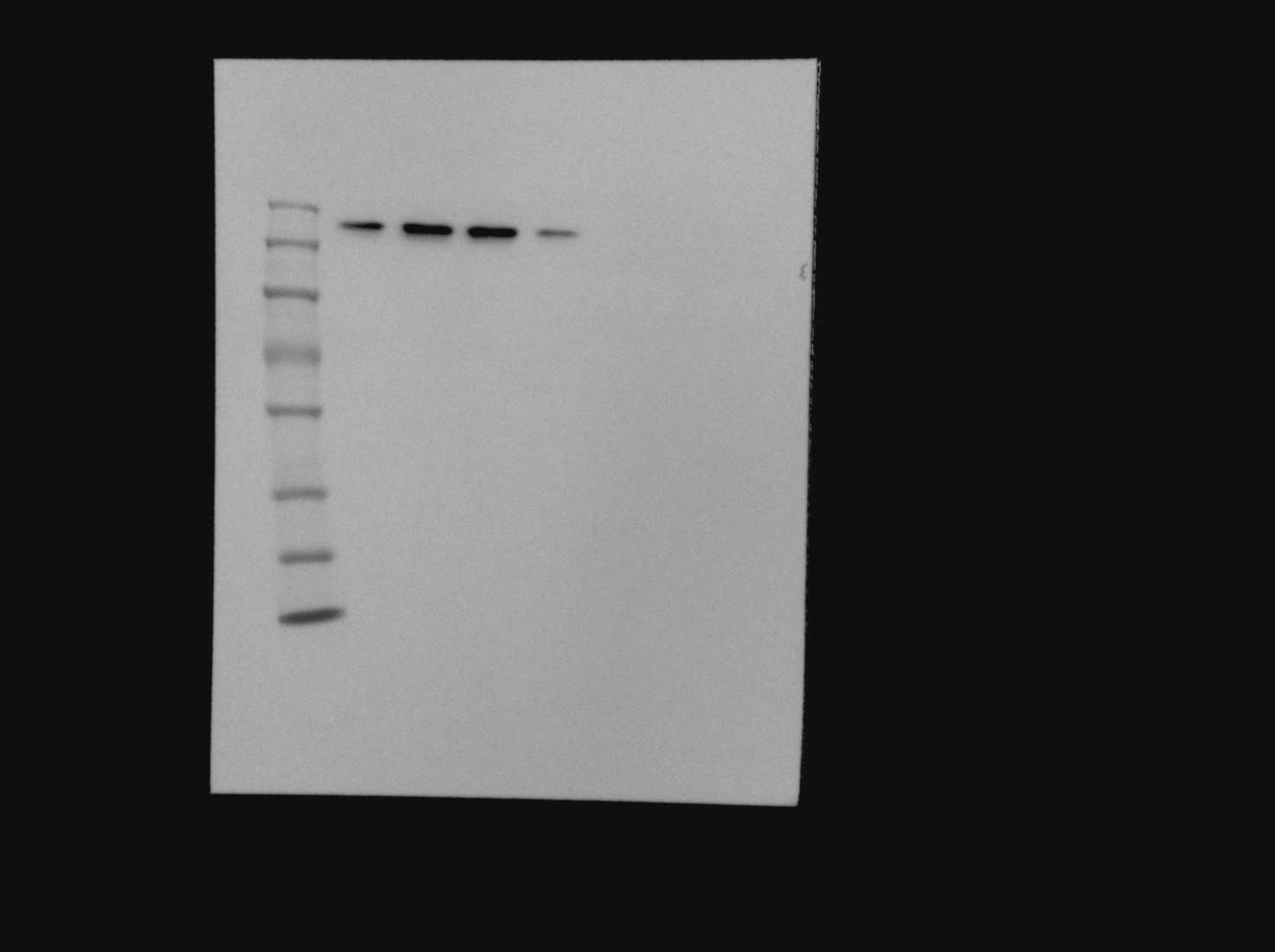


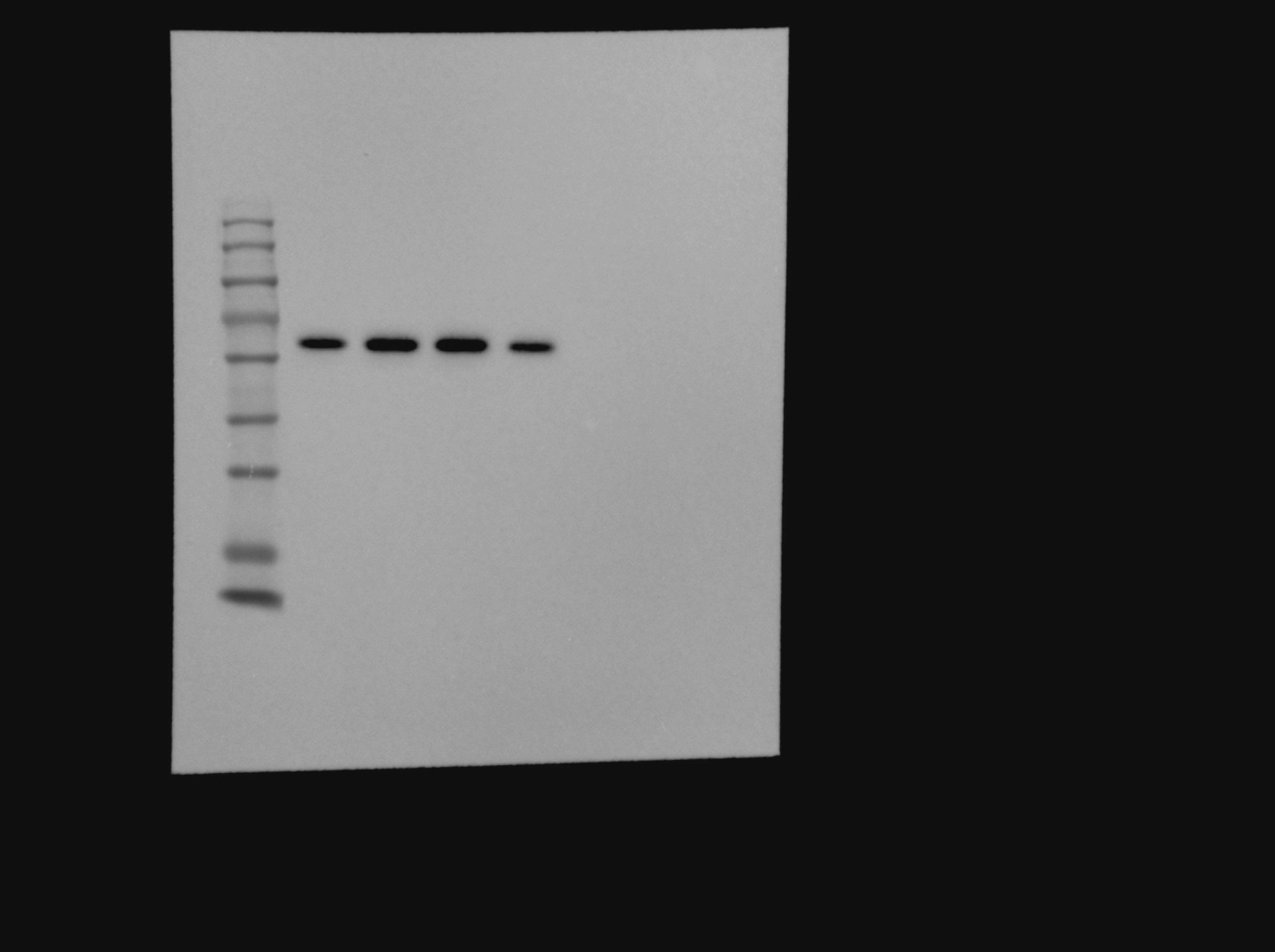


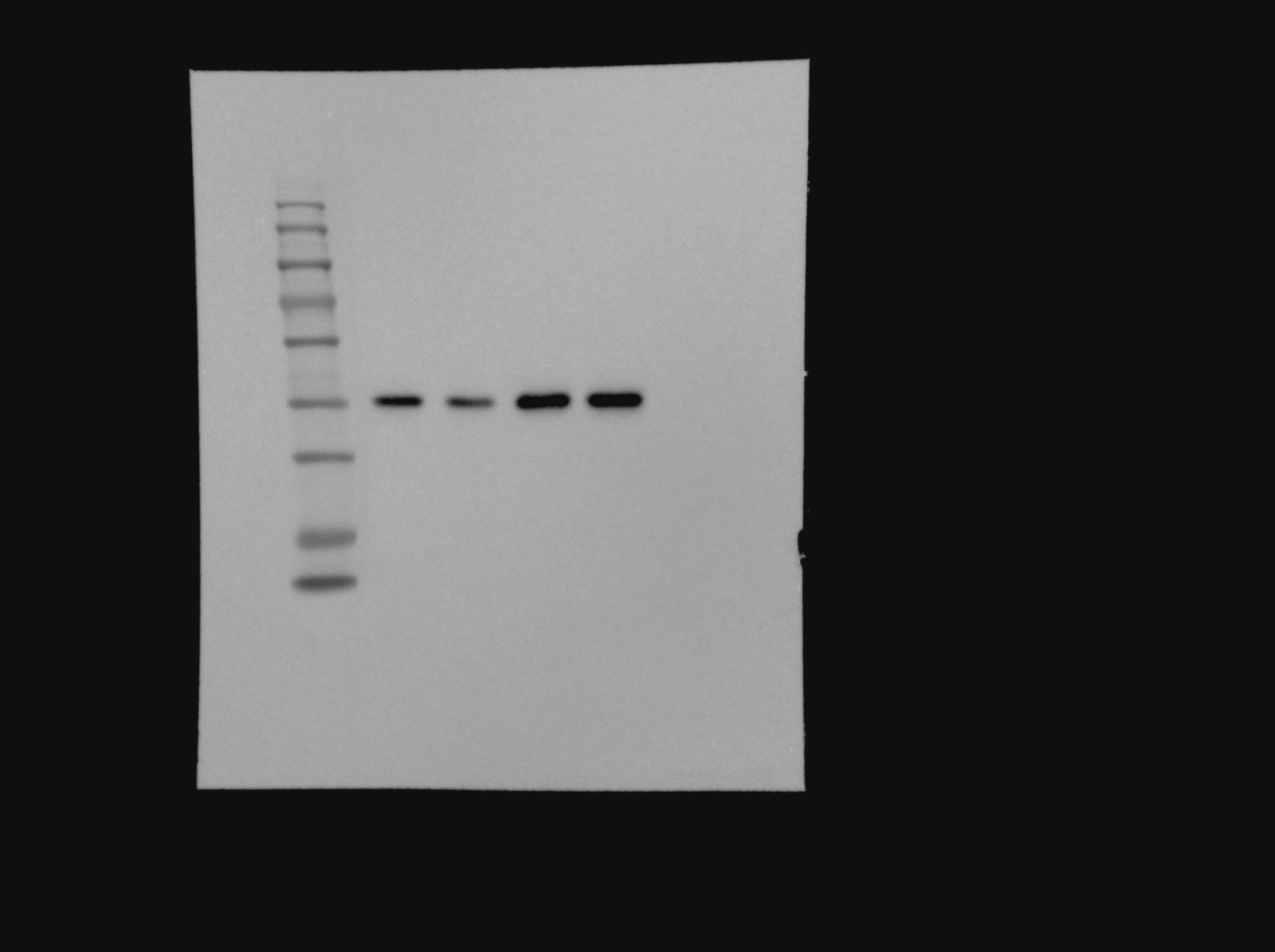


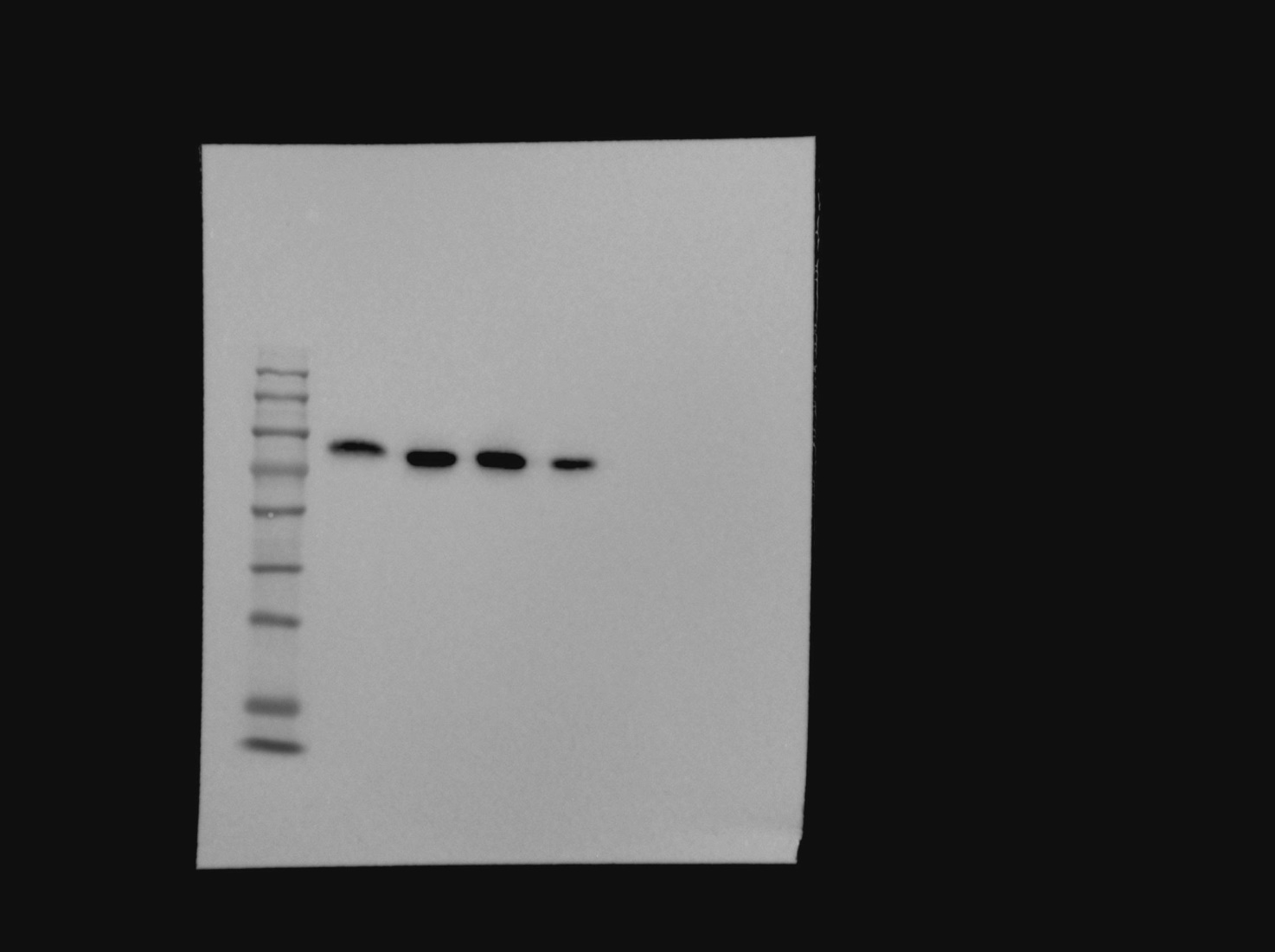


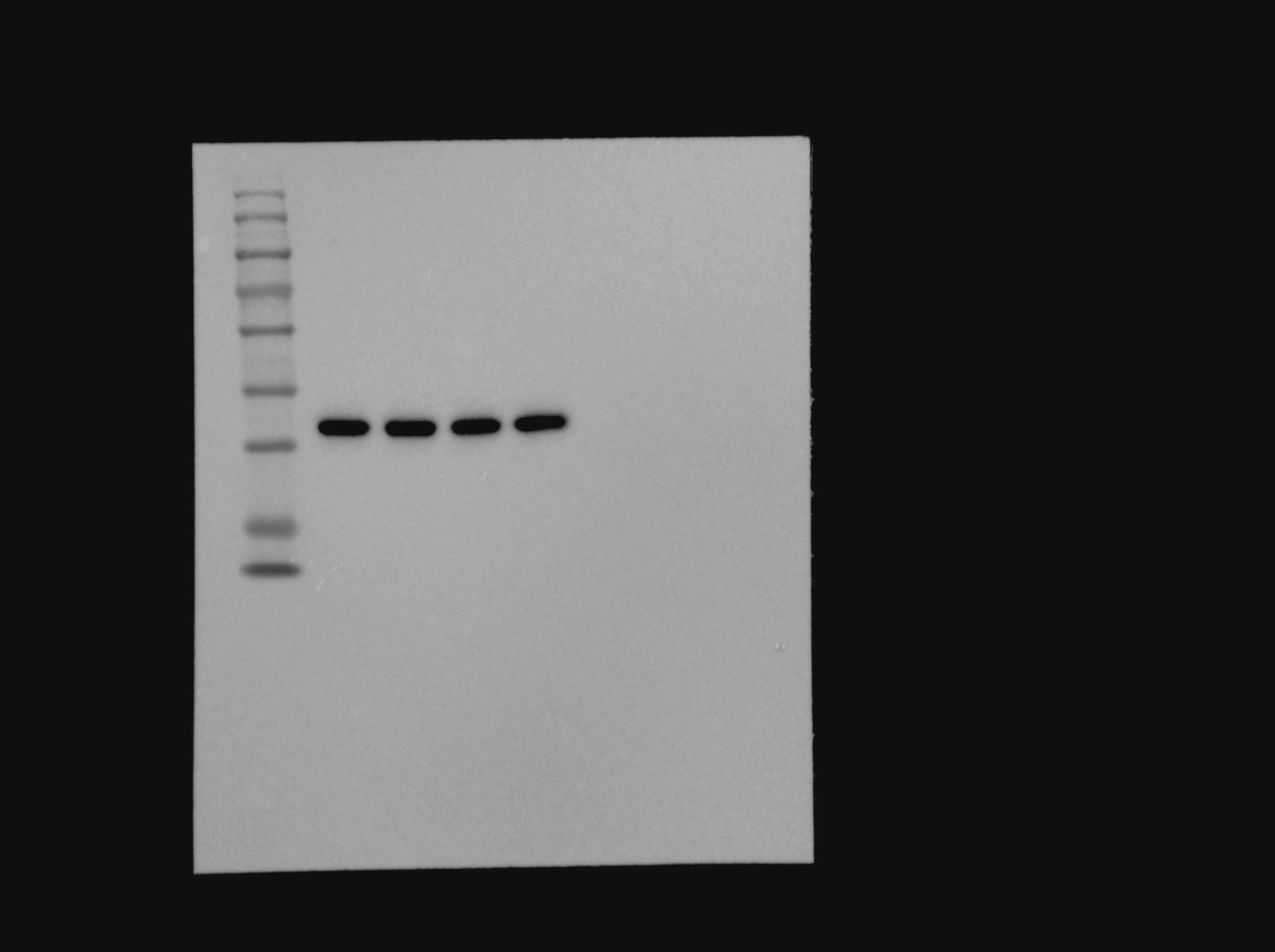

Supplement: Supplementary file 2 — original western blots [file 41420_2022_1179_MOESM2_ESM.docx]
